# Defining Symptom Concepts in Chronic Subjective Tinnitus: Web-Based Discussion Forum Study

**DOI:** 10.2196/14446

**Published:** 2020-01-07

**Authors:** Alice Hibbert, Markku Vesala, Micky Kerr, Kathryn Fackrell, Stephen Harrison, Harriet Smith, Deborah Ann Hall

**Affiliations:** 1 Hearing Sciences Division of Clinical Neuroscience, School of Medicine University of Nottingham Nottingham United Kingdom; 2 National Institute for Health Research Nottingham Biomedical Research Centre Nottingham United Kingdom; 3 Tinnitus Hub Ltd Hemsworth United Kingdom; 4 University of Nottingham Malaysia Semenyih Malaysia

**Keywords:** patient outcome assessment, treatment outcome, concept formation, qualitative research, patient participation, community participation, stakeholder participation, Web social networking

## Abstract

**Background:**

A minimum standard based upon consensus decision making recommends a core set of tinnitus-specific health complaints (outcome domains) that should be assessed and reported in all clinical trials as this enables comparisons to be made across studies as well as data pooling for meta-analysis.

**Objective:**

This study aimed to further clarify how the outcome domain concepts should be defined for 5 of the core set: tinnitus intrusiveness, sense of control, acceptance of tinnitus, concentration, and ability to ignore. This step requires a clear and fully elaborated definition for each outcome domain, moving from an abstract or a vague concept to an operationalized and measurable health-related construct, so that a suitable measurement instrument can then be identified.

**Methods:**

A series of 5 focus group–style semistructured discussions were conducted via a Web-based discussion forum, each open for 2 weeks and ending with a vote. The participants included 148 tinnitus experts who completed a preceding e-Delphi survey that had generated the original set of minimum standards. The participants were health care users living with tinnitus, health care professionals, clinical researchers, commercial representatives, and funders.

**Results:**

The Web discussions led to a revision of all 5 original plain language definitions that had been used in the preceding e-Delphi survey. Each revised definition was voted by 8 to 53 participants and reached the prespecified threshold of 70% consensus for all except tinnitus intrusiveness. Although a single definition was not agreed upon for tinnitus intrusiveness, the majority of participants shared the view that the concept should be sufficiently broad to encapsulate a range of subdomains. The examples included tinnitus awareness, unpleasantness, and impact on different aspects of everyday life. Thematic analysis of the 5 Web-based discussion threads gave important insights into expert interpretations of each core outcome domain, generating an operationalized and measurable health construct in each case.

**Conclusions:**

The qualitative data gathered during the Web-based discussion forum provided an important in-depth understanding of the health concepts that had raised a debate during earlier face-to-face meetings. The descriptive summaries and definitions provide sufficient operationalization of those concepts to proceed to the second stage of core outcome set development that is to identify and evaluate suitable measurement instruments. This study supports the use of Web-based peer discussion forums in defining health concepts.

## Introduction

### Background

Chronic subjective tinnitus is a condition characterized by a persistent auditory sensation (eg, ringing, whistling, hissing, and buzzing) experienced only by the individual, with no corresponding external sound or source. The characteristics and impacts of tinnitus are highly variable from person to person [1], and the outcomes reported in clinical trials of tinnitus interventions are similarly diverse [2]. This prevents the comparison of findings across trials and pooling data in meta-analyses, leading to a waste of research resources and an unreliable evidence base for making decisions about which interventions are most effective [3].

The development of core outcome sets (COSs) can tackle this issue by establishing a common standard and minimum set of recommended core outcomes for use in clinical trials of a specific condition or intervention type as well as for use in other types of research and clinical audit [4]. The Core Outcome Measures in Effectiveness Trials (COMET) handbook [5] outlines guidelines for best practice in COS development and advocates a 2-step approach to COS development. The first step considers *what* condition-related complaints should always be collected and reported. In this paper, the *what* is henceforth referred to as an *outcome domain*. Once an agreement has been reached regarding what should be measured, *how* those outcomes should be measured is then determined [6]. This 2-step process has the advantage of being able to define each outcome domain so that it is understood by patients and clinicians in a consistent way and also to identify gaps where further research would be needed, for example, if an outcome domain is seen to be of core importance but no adequate outcome measurement instrument yet exists.

### Research Leading up to This Study

**Figure 1:**
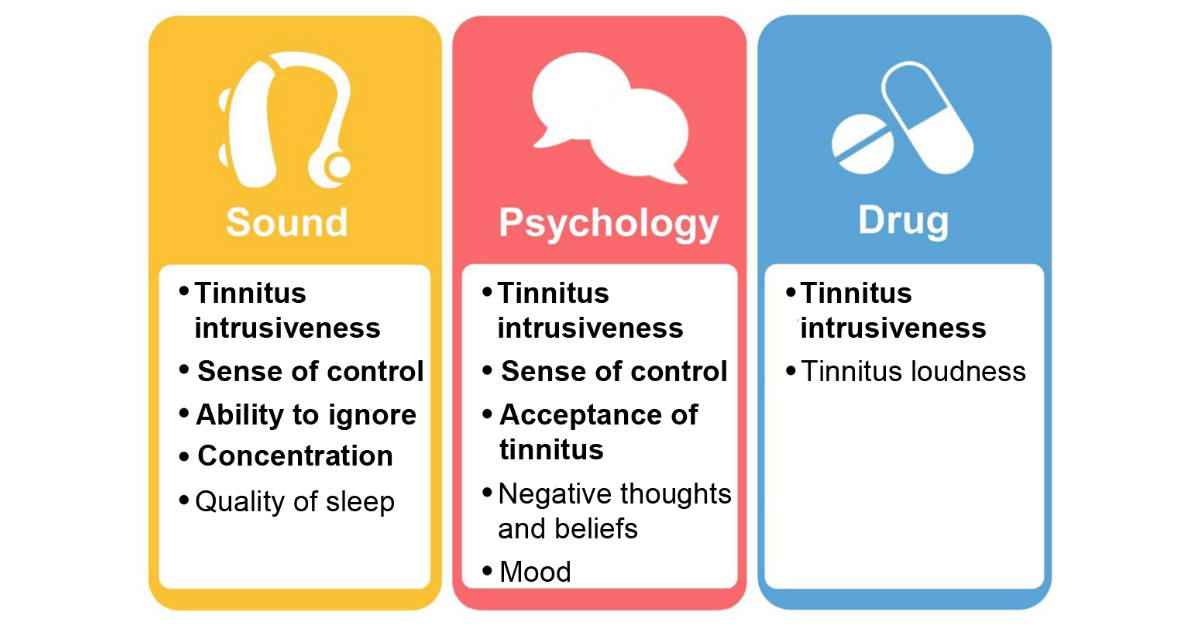
Graphic illustrating the Core Outcomes Measures in Tinnitus: International Delphi recommendations for core outcome domain sets for each family of interventions widely available for chronic subjective tinnitus in adults. Core outcome domains highlighted in bold are those considered in detail within this paper and represent 5 out of the 9 distinct domains.

The research reported in this paper is part of a longer-term program by the Core Outcomes Measures in Tinnitus (COMiT) initiative that aims to establish a COS for clinical trials assessing interventions for chronic subjective tinnitus in adults [7]. The first step of COS development has recently been completed by the Core Outcome Measures in Tinnitus: International Delphi (COMiT’ID) [7-12] study. This first step developed minimum recommendations for what all tinnitus clinical trials should measure. The methods included a series of international electronic Delphi surveys and face-to-face consensus meetings in which core outcome domains were defined for the 3 most common tinnitus intervention types: sound, psychology, and pharmacology-based approaches [11,12]. A total of 73 candidate outcome domains were considered during this process and, for each outcome domain, a plain language concept definition was cocreated with patient and public representatives via qualitative methods [8]. These domains and definitions were evaluated by 719 stakeholders with expertise in tinnitus, including both health care users and professionals [10]. The result was an agreement on 9 distinct core outcome domains across the 3 intervention types [11], which is summarized in Figure 1 (adapted from [11]). These core outcome domains were then ratified through Web (email) votes opened to all of the original electronic Delphi survey participants [11].

### Defining Symptom Concepts

In accordance with the COMET handbook [5], the second step of COS development is to identify how each core outcome domain should be measured. This step requires a clear and fully elaborated definition for each, moving from an abstract or a vague *concept* to an operationalized and a measurable *construct*. This is emphasized by the consensus-based standards for the selection of health measurement instruments initiative [13] that explains: “When selecting an outcome measurement instrument for research or clinical practice, first the outcome to be measured should be clearly defined... For example, when measuring a broad construct such as health-related quality of life, it should be clarified which subdomains are relevant for the target population in the specific context of interest. Sometimes several definitions exist for an outcome…Without explicitly defining or describing the intended outcome, people may have different ideas about it and interpret it differently.” A detailed definition of the construct based on the specific area of health to which the core outcomes are to apply is a prerequisite for selecting an appropriate outcome measurement instrument [6]. The definitions and interpretations of 4 of the 9 core outcome domains (*quality of sleep, mood, negative thoughts and beliefs,* and *tinnitus loudness)* reached agreement by health care users and professionals during the COMiT’ID consensus meetings and so did not require further exploration and elaboration [11]. Reviewing those meeting discussions was considered sufficient to consolidate and finalize the definitions and conceptualizations of those 4 core outcome domains (see Multimedia Appendix 1). In contrast, those same discussions had highlighted the need for further work to specify and define the remaining 5 core outcome domains. First, it became evident that there were individual differences in the personal meaning attributed to certain domains and a lack of consistency in how they were understood by all stakeholders. Second, respondents made decisions to support certain outcome domains on the condition that their definition would be expanded to capture other outcome domain concepts (as subdomains). The purpose of this study was to specify and define these remaining 5 core outcome domains.

This study used some important new knowledge relating to the 5 core outcome domains of interest that had been gathered during the previous stakeholder discussions [11,12]. This can be summarized as follows: For *tinnitus intrusiveness*, there were a diverse range of interpretations about precisely what the concept entails. *Tinnitus intrusiveness* is commonly measured using a single-item numerical rating scale in which the concept is not defined [2]. Attempts to measure *tinnitus intrusiveness* as a construct using multiple questions assess a range of subdomains such as tinnitus awareness, loudness, unpleasantness, annoyance, and ability to ignore [14,15]. There was some discussion about potential negative connotations and misinterpretations of both *acceptance of tinnitus* and *sense of control*. These domains were often discussed together and compared with one another, but the exact relation and association between the 2 was unclear. Similarly, *ability to ignore* and *concentration* were often thought of as interacting with each other and were considered broad concepts that captured the essence of some of the other outcome domains that had been set aside from the core set. Furthermore, differing viewpoints emerged about whether *ability to ignore* should refer to change in the tinnitus itself or refer to an individual’s personal capabilities. Resolving these debates is required to reach a common understanding of each construct so that it can be operationalized and mapped onto appropriate measurement instruments to ensure that the instrument has good content validity [6].

Currently, there are no formal guidelines to assist COS developers on how to further conceptualize and define outcome domains [5,6], and so COS developers have proceeded using different methods. For example, the World Health Organization’s International Classification of Function, Disability, and Health has been used as a general reference framework for conceptualizing health-related quality of life in chronic pain [16] and in rheumatic conditions [17]. To help define atopic eczema flares, the Harmonising Outcome Measures for Eczema initiative first conducted systematic reviews of the literature [18,19] followed by a statistical evaluation of the performance of instruments measuring 2 alternative definitions of atopic eczema flares [20]. The first method relies upon symptoms being linked to concepts in the reference framework, whereas the second relies on a body of literature assessing the construct of interest. Neither method is suitable for chronic subjective tinnitus, where the core outcome domains of interest do not map well onto the World Health Organization’s framework [1] and are not represented by an adequate body of literature [2].

### Web-Based Discussion Forums in Core Outcome Set Development Work

Although the uptake of Web discussion forums as a platform for COS development is somewhat in its infancy, they have been successfully applied to evaluate the face validity of a new patient-reported outcome measure of treatment response in vitiligo [21], to explore patient perceptions of proposed core outcome domains for eczema [22,23], and to investigate patient priorities for a COS for pediatric acute respiratory illness [24]. Web-based platforms are now growing in popularity within the tinnitus research community, and self-help discussion forums are starting to be used for recruitment [25] and research data collection [26].

### Aim

In summary, the aim of this study was to specify and define the 5 least well-defined core outcome domains recommended for clinical trials evaluating the effect of sound-, psychology-, and drug-based tinnitus interventions. The 5 core outcome domains in need of discussion were (1) tinnitus intrusiveness, (2) sense of control, (3) acceptance of tinnitus, (4) concentration, and (5) ability to ignore, and these were to be explored using a moderated Web discussion forum with representative stakeholders from the COMiT’ID study. The goals were to establish agreement on a single plain language definition describing each of the core outcome domains and to gain a more in-depth understanding of each concept that would then indicate what sort of questions would need to be asked when assessing each outcome.

## Methods

### Design

This qualitative study used a series of 5 focus group–style discussions conducted via a Web discussion forum. The Web discussion forum was chosen as a practical and cost-efficient research method for engaging with a large and geographically distributed sample of participants, which could not be achieved by face-to-face methods.

Each Web discussion focused on a single core outcome domain that included *tinnitus intrusiveness*, *sense of control*, *acceptance of tinnitus*, *concentration*, and *ability to ignore*. The discussions were semistructured and ended with a voting phase that was focused on the executive summary of the discussion and the resulting concept definition, following recommendations by Im and Chee [27].

This study was conducted under a substantial amendment to the ethical approval originally granted for the COMiT’ID electronic Delphi and consensus studies by the West Midlands—Solihull Research Ethics Committee and Health Research Authority (reference 17/WM/0095, March 2017). This amendment was approved on September 18, 2017.

### Recruitment and Participants

All registered COMiT’ID participants were invited by email to join the Web discussion forum. We had taken a number of steps to safeguard the relevant expertise (and hence representativeness) of these participants, and the details are published elsewhere [8,12]. We did not contact those who had explicitly withdrawn, and so 627 individuals were invited from a total of 641 unique individuals who had registered [10]. Regular updates via twitter [28], direct email, and at the international Tinnitus Research Initiative conference in March 2018 continued to encourage registration throughout the study.

The invitation and reminder emails contained a link to the discussion forum website [29] and a verification code that was required to register an account on the website. The code maintained privacy and security, ensuring that only those individuals who registered for the electronic Delphi survey were able to access the forum.

Eligible participants included members of the public with lived experience of tinnitus, health care practitioners, clinical researchers, and commercial representatives or funders. All the participants were targeted using a purposive sampling approach and had signed a self-declaration statement confirming that they met our eligibility criteria for having expertise on tinnitus. For full details see previous studies by Hall et al [11,12].

### Procedure

#### Design of the Web-Based Discussion Forum

The website for the discussion forum was developed in partnership with the Tinnitus Hub [30]. Tinnitus Hub is a nonprofit organization that provides peer-to-peer support for those living with tinnitus and connects health care users with professionals conducting research. Tinnitus Hub hosts a peer support forum called Tinnitus Talk [31] that is one of the largest international tinnitus discussion forums and was selected for our research forum for its widespread reputation, secure platform, anonymity, and proven track record of engagement by the tinnitus community. This latter reason is particularly important given that people with tinnitus are older adults [32] who may be less familiar or comfortable with using Web discussion forums [33].

The Tinnitus Talk platform offered a number of positive design features well suited to the research aims and encouraging active engagement in the discussion. The participants could be individually distinguished, but their anonymity was preserved through the use of a pseudonym rather than their true name. The posts were automatically ordered chronologically, which allowed the discussion to be read as a conversation. A direct reply feature quoted the original post, avoiding the need to scroll back and forth through the discussion, and sent a notification to the author of the original post, nudging them to return to the forum and encouraging a natural flow back and forth, similar to face-to-face conversation. The participants could not alter their responses after posting but could add further comments to clarify or change their opinions.

Instructional videos were created to improve usability regardless of experience in Web discussion forums and technical ability. An introductory video on the homepage [29] guided participants through how to register and create an account (see Multimedia Appendix 2). Once logged in, a second video guided participants on how to write posts, reply to others, and set email notifications (see Multimedia Appendix 3).

Overall, 3 informational threads were open throughout the study: (1) an *Introduction,* which set out how the forum would run, with recommendations on how to take part, (2) *Guidelines and Ground Rules,* which stipulated rules such as respect for one another’s opinions and expertise, and (3) *Tech Support FAQ,* which provided advice and solutions for common technical problems that might be encountered while using the Web forum. All participants were encouraged to read these threads and post a reply to practice using the forum software interface and to confirm that they had understood and agreed to follow the ground rules.

**Figure 2:**
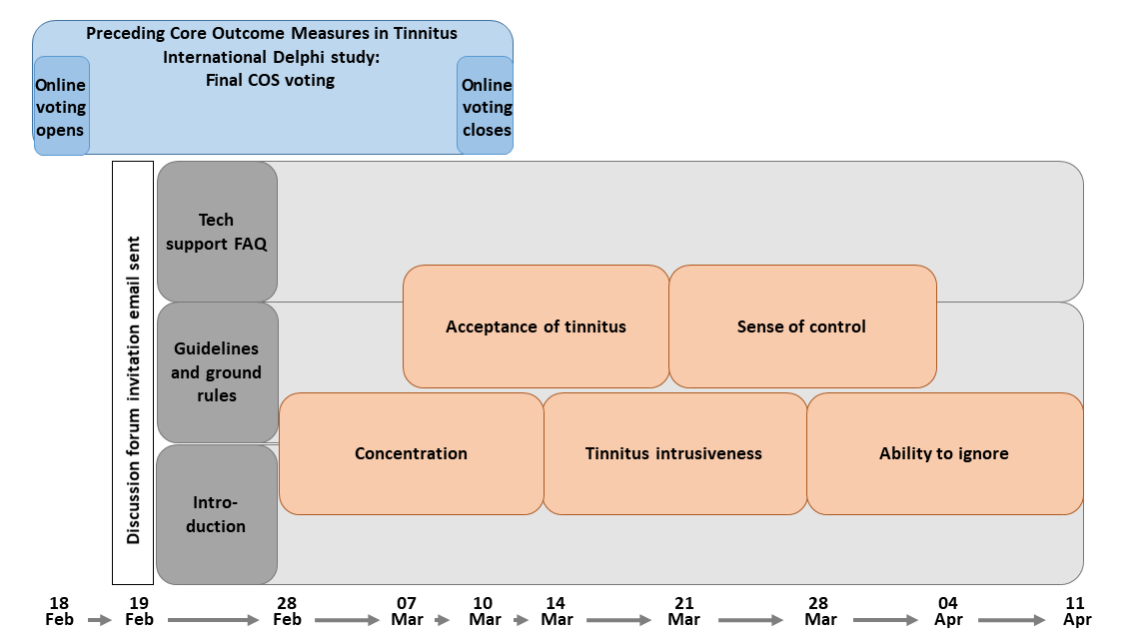
Timeline illustrating the design of the Web discussion forum, with dates indicating the duration of each discussion topic. The Core Outcome Measures in Tinnitus International electronic Delphi survey was in its final (voting) stage at the launch of the forum. FAQ: frequently asked questions; Feb: February; Mar: March; Tech: technology.

In addition, 5 further threads were used for each of the 5 focus group–style discussions, one for each core outcome domain (Figure 2). Individual threads overlapped in time so that the total discussion period was 6 weeks (Figure 2). All discussion threads were always visible, but they remained locked until the advertised opening date. The first discussion thread opened 2 weeks after the invitation email. Thereafter, 1 discussion thread opened each week. Discussion threads were open for 2 weeks, with semistructured discussion over 10 days and then a moderator-led summary and voting. Individual threads were purposefully ordered according to our expectation that engagement would be greatest during the middle of the study period. Therefore, we chose to place those outcome domains that had generated the widest debate across weeks 3 and 4.

#### Methods for Reaching Agreement

All 5 discussion threads started with a reminder of the plain language definition for the outcome domain given during the electronic Delphi survey [8,9]. Most of the 2-week period comprised the semistructured discussion (see Multimedia Appendix 4) with a series of questions and discussion prompts that followed the natural flow of the conversation as much as possible. Throughout the semistructured discussion, participants were encouraged to not only answer the questions and discuss the concepts but also to suggest revisions for the plain language definitions.

The moderator then gave a brief executive summary of the discussion and proposed a final revised plain language definition. Wherever possible, the revised plain language definition was proposed, refined, and supported by participants during the discussion. Where this was not possible, the moderator developed a revised plain language definition based on the key themes raised during the discussion. These key themes were identified by a preliminary qualitative analysis of the discussion content conducted by the independent moderator as the discussion was unfolding.

Participants were asked to cast their vote according to 4 options: (1) agree with both the summary and definition, (2) agree with the summary but not the definition, (3) agree with definition but not the summary, and (4) disagree with both the summary and definition. Those who disagreed were asked to explain their reason and to recommend any changes. The Web platform used for the forum restricted voting to holding 1 per discussion thread. So, in cases where there was more than one plain language definition, participants were asked to choose their preferred definition. Any disagreements with the summary were collected in the form of written viewpoints instead of an actual vote. Consistent with the preceding electronic Delphi survey, at least 70% agreement across respondents was considered the threshold for accepting the summaries and definitions [5,8].

#### Moderation Style

Each discussion was led by an independent moderator (AH) who had experience in leading focus groups and had not been involved in earlier stages of COMiT initiative work or experienced tinnitus herself. However, she did undergo a period of familiarization with the work conducted to date, including listening to recordings of the face-to-face consensus meetings. The moderating style was flexible, becoming more or less active depending on the degree of participant engagement. The aim was to foster a natural conversation style between participants as would happen during a face-to-face focus group. The most desirable style of conversation was one in which participants clearly explained and responded to one another’s personal perspectives to reach a shared understanding. Posting of monologues or isolated messages to the moderator was discouraged. To promote this desired style of discussion, the moderator posed carefully worded (ie, nonleading) questions to encourage participants to elaborate and to give specific examples where more detail would be useful. The moderator also regularly reemphasized the key questions and topics to cover, reinforcing and thanking participants for their contributions and bringing them into conversation with one another on occasions when they had shared either similar or contrasting perspectives. When disagreements or tension arose, the moderator reminded participants of the purpose and context of the discussion forum and the ground rules.

The moderator also maintained a high degree of contact with participants to prompt and remind them of the structure and next stages of the procedure. For example, reminder emails were sent to highlight the next discussion thread opening and to encourage voting before a discussion thread closed. In response to participant feedback, the moderator updated the first post in each discussion thread so that it summarized each key question asked in the forum, with hyperlinks to the corresponding post. The intention was to ease the burden on participants, allowing them to make informed contributions without necessarily needing to read every single post in the thread.

### Analysis

#### Executive Summary and Plain Language Definition

The first stage of the qualitative analysis relating to *tinnitus intrusiveness*, *sense of control*, *acceptance of tinnitus*, *concentration*, and *ability to ignore* was to generate a brief executive summary and, if needed, to revise the plain language definition for each of these outcome domains. These outputs were generated while the discussion thread was open so that they could be used in the Web-based voting phase.

The moderator (AH) prepared each executive summary to address 3 aspects of the discussion. One aspect of the discussion concerned key concepts that were discussed and new themes that emerged. The moderator determined what was key based upon themes that were most often mentioned and talked about by participants, and what appeared to be most relevant to the study aim. The second aspect concerned any recurring themes or strongly dissenting concerns that might necessitate a revision to the plain language definition. The third aspect concerned views where a concept for one core outcome domain seemed to converge with another concept that had previously been set aside from further discussion (either during the electronic Delphi survey or consensus meeting) [11]. The executive summary was written in such a way that a vote of agreement indicated support for all 3 aspects. These 3 discussion points might reasonably necessitate a revision to the plain language definition. Where possible, the revised plain language definition used phrases given by the participants. In cases where participants had offered multiple definitions, the moderator selected the one that appeared to reflect the majority’s viewpoint. If this was not possible, then all of the candidates were asked to vote.

#### In-Depth Understanding of Each Concept

In addition to the moderator-led executive summary, thematic analysis was applied to a download of the entire 5 discussion threads conducted by 2 analysts (AH and MK). MK provided an independent perspective as he was naïve to the project. The 2 analysts independently examined each discussion thread separately and, in the order, that they took place. Methods for thematic analysis followed Braun and Clarke’s 6-stage framework [34], and qualitative analysis was conducted using NVivo Pro 11 software (QSR International Pty Ltd, version 11). The text was read several times for familiarization before it was coded. Emerging themes were identified by grouping codes, and they were then refined and defined through an iterative process. Coded text segments provided the evidence corresponding to each theme. Once they had completed this process independently, the 2 analysts met together with the principal investigator (DAH) to compare their independent analyses and codebooks, with the intention of identifying and validating key themes to be reported for each outcome domain. To achieve this, similar themes were merged, any discrepancies were resolved, and those themes most relevant to the study objectives were identified.

## Results

### Participants and Engagement

Of the 627 individuals invited, 251 registered for the Web discussion forum, leading to a recruitment rate of 40.0% (251/627). Of these 251 participants, 119 submitted one or more posts to the discussion forum. Henceforth, these are referred to as *discussants*. An additional 29 participants did not submit any posts but took part by voting in at least one of the discussion threads, leading to an engagement rate of 59.0% (148/251). To preserve anonymity, we did not request information about stakeholder group membership. However, many discussants freely disclosed their stakeholder affiliation in the content of their posts. Although these data are indicative not definitive, 53.7% (64/119) participants identified themselves as health care users living with tinnitus and 25.2% (30/119) as professionals, and the remaining 21.0% (25/119) were unknown.

Similar degrees of activity were sustained across the 5 discussion threads (Table 1). Inevitably, some discussants were more active than others, but no individuals sought to dominate the conversation. Some held strong personal viewpoints, for example, “The reason I feel strongly about the negative impact side of intrusiveness is from my personal experience of tinnitus.” However, these tended to be internally regulated by the forum discussants, for example, “I don't think you can compare one set of experiences to another fully because of the wild variety of coping strategies that people will use.” Although the number of posts and discussants was broadly equivalent across the 5 discussion threads, the number of voters was markedly low for *concentration*, perhaps because it was the first thread and participants were still familiarizing themselves with the structure of the forum discussions and with the limited time window available for voting. The number of voters were also relatively low for *tinnitus intrusiveness*. The possible reasons for this are less clear, but several speculations can be offered. For example, the voting options for tinnitus intrusiveness required participants to choose between several potential definitions rather than simply agree or disagree with a single definition, and this different voting process may have been less appealing or confusing to participants. For example, 3 discussants very active in the forum when the vote was open preferred to share their view by submitting multiple written posts that commented on the voting options available rather than casting a vote.

**Table 1:** Participant activity across the 5 discussion threads evidenced by the total number of posts, number of unique individuals who submitted posts (discussants), and the number of unique individuals who voted (voters). Note that the number of posts includes those submitted by the moderator.

Participants (type)	Number of participants in each discussion thread				
	Tinnitus intrusiveness	Sense of control	Acceptance of tinnitus	Concentration	Ability to ignore
Posts	160	133	147	142	145
Discussants	49	44	54	49	47
Voters	20	33	53	8	44

**Figure 3:**
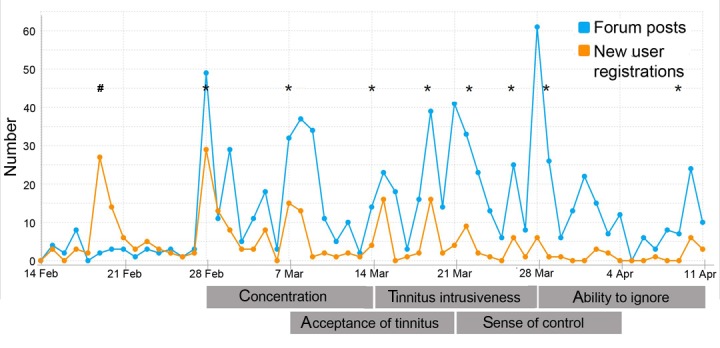
Graph showing the number of user registrations and forum posts over the 8 weeks, from the initial invitation email to closure of the final discussion thread. The hashtag symbol (#) indicates the first mass invitation email, and asterisk symbols (*) indicate subsequent reminder emails. Reminders generally coincided near the start and midway through each discussion thread but were required less toward the end of the study. Apr: April; Feb: February; Mar: March.

The level of activity on the Web discussion forum is plotted in Figure 3 across the relevant 8-week period from the first email invitation being distributed to the closure of the last discussion thread. This displays that engagement was sustained fairly consistently throughout the procedure and demonstrates the benefit of sending reminder emails and regular updates via other social media.

### Executive Summaries and Plain Language Definitions

The executive summaries created from each Web discussion forum are presented in Multimedia Appendix 5, and the plain language definitions and percentage of supporting votes are presented in Table 2. Table 2 also includes expansions to each concept with regard to which subdomains should be included in the operational definition, as recommended by participants in the discussion forum. Subdomains denoted as *(maybe)* are those suggested by a few of the discussants only and so are not critically important to include in the operational definition.

In the preceding consensus meetings, what constitutes *tinnitus intrusiveness* had generated the widest debate, and this was also true for the Web discussion forum. Several different versions of a definition were proposed but none seemed to gain majority support. A total of 7 alternative versions were put to the Web vote, but none reached the required 70% agreement to be able to conclude that a consensus had been reached. These different views on what tinnitus intrusiveness means for a person with tinnitus were consistent with the numerous suggestions to broaden its description to encapsulate other domain concepts such as the impact on different aspects of life, unpleasantness, and annoyance (Table 2).

It was a recurring theme during both the consensus meetings discussing sound-based and psychology-based interventions that *sense of control* and *acceptance of tinnitus* might converge onto a similar concept, along with the set aside domain *coping*. The Web discussion forum gave greater clarity on the distinction between these 2 core outcome domains. There was consensus that *sense of control* refers to the feelings achieved once a treatment or coping strategy has been found that provides relief, whereas *acceptance of tinnitus* refers to the general feeling of being at peace with the tinnitus and no longer fighting against it. Some of the discussants indicated a temporal order whereby *acceptance* might follow the (re)gaining of a *sense of control*. Although these 2 concepts are undeniably related, the definition for a *sense of control* perhaps encapsulates a situation-specific feeling that is related to actively managing the tinnitus, whereas the definition for *acceptance of tinnitus* encapsulates a more general feeling about letting go of the resistance to and distress caused by the tinnitus.

**Table 2:** The plain language definitions for each of the final core outcome domains, and percentage of voters who supported the revised plain language definition where this was changed during the course of the Web discussion forum.

Outcome domain	Original plain language definition	Revised plain language definition	Number of agreements among voters, n (%)	Subdomains
Tinnitus intrusiveness (n=20)^a^	Noticing the sound of tinnitus is there and it is invading your life or your personal space	The extent to which tinnitus invades your life, stresses you in daily situations and prevents you from doing things you want to do The unacceptable and unwelcome interference of internal head and body noise heard only by the individual Being acutely aware of the sounds of tinnitus, feeling that it is invading your life or your personal space, changing your thoughts or actions and negatively impacting on your life	7 (35) 3 (15) 4 (20)^b^	Tinnitus awareness Tinnitus unpleasantness Impact on individual activities Impact on social life Impact on relationships Impact on work (maybe) Annoyance (maybe)
Sense of control (n=33)^a^	Whether or not you feel you have a choice in how to manage the impact of tinnitus and feelings caused by tinnitus	Feeling that you have effective options for managing the impacts of and feelings caused by tinnitus, through an understanding of your condition, learned strategies, and/or available resources	27 (82)	Coping
Acceptance of tinnitus (n=53)^a^	Recognizing that tinnitus is a part of your life without having a negative reaction to it	Recognizing that tinnitus is a part of your life and staying neutral toward it in both thoughts and actions	44 (83)	N/A^c^
Concentration (n=8)	Ability to keep your attention focused	The ability to keep your attention focused on whatever you wish	8 (100)	Impact on work (maybe)
Ability to ignore (n=44)	Ability to continue as normal as if tinnitus were not there	The ability to continue as if tinnitus were not there	35 (80)	Conversations Listening

^a^Number of voters.

^b^Synthesized from comments made by 4 (20) voters who could not choose between the given definitions.

^c^Not available.

Similarly, the Web discussion forum gave greater clarity on the distinction between *concentration* and *ability to ignore*. There was consensus that *concentration* refers to the ability to focus on a specific task or activity that requires full attention, whereas *ability to ignore* describes the capacity to focus away from tinnitus in most if not all situations. Some of the discussants suggested that *ability to ignore* may be more relevant to low-demand tasks, whereas *concentration* may be more relevant to high-demand, complex tasks.

### In-Depth Understanding of Each Health Concept

The themes emerging from the discussion thread under each core outcome domain are reported in this section (but in no particular order).

#### Tinnitus Intrusiveness

Overall, 2 major themes emerged from the discussion thread: one highlighting the importance of the negative impact of tinnitus on everyday functioning and another highlighting the strength of its negative emotional impact.

#### Negative Functional Impacts of Tinnitus, Not Just Awareness of Its Presence

A prominent debate concerned whether the concept of *tinnitus intrusiveness* should reflect the ongoing presence of tinnitus and the individual’s awareness of it, or whether it should go beyond this to capture the negative impacts of tinnitus on everyday life. Although the discussants’ initial preferences seemed divided, by the end of the discussion thread, the overwhelming majority of posts supported the latter interpretation. For example, to argue against awareness of the presence of tinnitus, one discussant said: “it’s not the tinnitus itself that’s the problem it’s the fact that it’s invasive and having invaded it causes problems—specifically that it makes doing certain things (sleeping, socialising, whatever) either less do-able or at least less enjoyable.” (This participant later stated that they had meant to use the word “intrusive” rather than “invasive”).

The course of the discussion was guided by the ultimate objective of measuring *tinnitus intrusiveness* to assess whether an intervention is effective. For example, to argue in favor of the negative impacts, 1 discussant said:

I know it’s possible to have noticeable but not disabling tinnitus... A treatment should aim to combat the negative effects. Begs the academic question of whether a treatment might work for people for whom tinnitus was never a problem, just something they noticed.

Consistent with this, discussants voiced support for *tinnitus intrusiveness* including facets of at least 3 other functional outcome domains (*impact on individual activities, impact on social life,* and *impact on relationships*). These had previously been agreed as critically important during the electronic Delphi survey, but had been set aside from the final core outcome domain sets. For example, 1 discussant wrote: “By its very nature, tinnitus can ‘intrude’ anywhere so the core outcome of ‘intrusiveness’ could quite easily apply to all of the impact outcome domains.”

#### Negative Emotional Impact and Its Potency

Another prominent theme concerned the emotional reaction to the intrusiveness of tinnitus. Discussants emphasized the potential for extreme suffering by describing how insufferable, intolerable, and invasive tinnitus could be. Different definitions of intrusiveness were drawn upon to help understand the depth of the concept, particularly exploring its personification. For example, 1 discussant wrote:

Intrusiveness itself is the inability to keep something unwelcomed be it physical or non-physical such as a thought, from infiltrating your mind/personal space without your permission. Tinnitus is like a burglar that enters your home, holds you hostage, but instead of stealing your belongings, it robs you of your sense of peace. It is therefore, intrusive and dominating.

Consistent with this, discussants voiced support for the inclusion of *tinnitus unpleasantness,* which had previously been agreed as a critically important domain during the electronic Delphi survey but had been set aside from the final core outcome domain sets. For example, 1 discussant wrote:

I am content with the definition of tinnitus intrusiveness covering all the impacts, unpleasantness and awareness. As I am writing I can hear my tinnitus very clearly. It is not unpleasant as such, but a nuisance I could do without. As I have already said at first my tinnitus was always unpleasant and at its worst I had to really concentrate on not giving way to panic. Those days have gone and I hope they never return.

To conclude, there was no majority agreement on a revised definition, but *tinnitus intrusiveness* was generally agreed by discussants to be a broad concept with subdomains, referring not only to the extent to which tinnitus has an unwanted presence and is deeply unpleasant but also the extent to which it negatively impacts upon daily life and activity. Hence, the construct *tinnitus intrusiveness* embodies both interference with functioning and psychological distress associated with tinnitus.

#### Sense of Control

Overall, 3 major themes emerged from the discussion thread: 1 highlighting the importance of autonomy in how an individual manages his/her own tinnitus, 1 highlighting the associated sense of empowerment, and 1 highlighting the importance of actively adopting management strategies.

#### Autonomy

The concept of free agency was related to the *sense of control*; the importance of an individual’s capacity to act independently and to make free choices and about how and when to apply management strategies. It was generally felt that this facet of *sense of control* required different options to be available and to be offered (by the health care professional). Autonomy was a phrase used by several discussants. For some, it was also important that the options were effective. It is important to note that making the choice *not* to do anything was considered equally relevant and acceptable. For example, 1 discussant wrote: “In some instances my tinnitus definitely defines some actions, entry into very loud shopping spaces and restaurants for instance are a step too far... Ear plugs help, but then leaving and being in control of that is a better choice. No [sic] convenient but better.”

#### Empowerment

A sense of free agency promotes self-efficacy, and so, one emerging theme emphasized the emotional consequence of feeling in control and being able to make personal choices. One example is the post: “When you are able to manage the impact/response to some extent and continue to enjoy life (by focusing on what you can do and reframing what you can’t) you overcome the helplessness and achieve some sense of control.” This positive sense of self was described by discussants as self-confidence, empowerment, and self-efficacy, whereas the converse was described as being at the mercy of tinnitus, leading to despair.

#### Active Management Strategies, Not Just State of Mind

Another emerging theme emphasized that *sense of control* should reflect a context in which the individual practices an active tinnitus management strategy. Some discussants expressed strong opinions that the concept of *sense of control* should not be restricted to the view that tinnitus suffering can be alleviated simply by changing negative thoughts or by adjusting one’s state of mind. One discussant said: “This control has to have been achieved as the result of an intervention—as others have said—it is not a ‘state of mind’ it doesn’t come about by ‘positive’ thinking.” Instead, it was agreed during the voting phase that *sense of control* should refer to an active, practical, autonomous approach to tinnitus, which may involve an array of interventions, strategies, treatments, tools, techniques, resources, and aids.

To conclude, discussants considered that *sense of control* referred to being in possession of active management options to cope with the impacts of and feelings caused by tinnitus. The revised definition was as follows: “Feeling that you have effective options for managing the impacts of and feelings caused by tinnitus, through an understanding of your condition, learned strategies and/or available resources.”

#### Acceptance of Tinnitus

Overall, 3 major themes emerged from the discussion thread: 1 highlighting the importance of handling negative reactions, 1 highlighting reconciliation of one’s own identity, and 1 emphasizing the ongoing struggle to maintain acceptance.

#### Becoming Less Reactive to Negative Reactions

The original plain language definition stipulated that *acceptance of tinnitus* was never having a negative reaction toward it. Some discussants felt this definition was “unrealistic...[and] inadequate in its understanding of the ups and downs of living with constant ringing in your ears,” particularly, those that interpreted not having negative reactions as implying the need for positive reactions. It was instead argued that a healthy level of acceptance is just getting better at managing and not responding to negative thoughts, bringing yourself back to a place of neutral balance, and choosing not to fight against the tinnitus. Useful comparisons were drawn to practicing meditation, acknowledging when your mind wanders, and calmly bringing it back to where you want it to be until it gradually becomes easier to do so and wanders less often. As 1 discussant stated, sometimes “it’s alright to have a negative reaction to tinnitus. Just let it be. It will pass.”

#### Self-Identity When Living With Tinnitus

During the discussion of *acceptance of tinnitus*, the theme of identity emerged as a complex and individualized issue. For some, recognizing tinnitus as a part of themselves was central to acceptance, whereas for others, it was important to distinguish between themselves and their tinnitus, maintaining their own identity as more than that. Compromise was found in the stance: “tinnitus is just small part of who you are but that it doesn’t define or control you.” This battle to rediscover and reconcile one’s own identity, classifying tinnitus as either internal or external to the self, seems integral to reaching acceptance, which was spoken about as “coming to terms with what is and not fighting against it,” and becoming able to “coexist with [their] tinnitus rather than see it as the enemy.” This seems to mark a turning point where those living with tinnitus begin to allow themselves to make empowered choices taking their tinnitus into consideration rather than struggling to always act in spite of it. One professional defined *acceptance of tinnitus* from their experience with patients as: “people choosing to explore living well even if tinnitus may be with them permanently.”

#### The Ongoing Struggle of Acceptance

Some discussants had very strong negative reactions to this concept, demonstrating hostility and frustration toward the idea of being told to *accept* their tinnitus which to them seemed completely intolerable or even harmful. To these people, notions of endurance, resilience, and tolerance seemed to be more conceivable milestones along the way. Even among those individuals who spoke more favorably of the concept of *acceptance of tinnitus*, it was clear that this is not something that is achieved and then easily maintained forever: “Acceptance is a process that comes and goes. It is a continuous process. The definition is too static.” It became apparent that any measurement of *acceptance of tinnitus* would have to understand and integrate an element of time to be accurate and recognize that those living with tinnitus “will have good days and bad days.”

To conclude, the revised definition of *acceptance of tinnitus* was “Recognising that tinnitus is part of your life, and staying neutral towards it in both thoughts and actions.” The concept was described by discussants as a highly individual and challenging experience, marked by one or many turning points by which achieving a sense of self-identity as someone living with tinnitus gives a greater sense of peace, and the struggle of living with tinnitus becomes somehow easier.

#### Concentration

Overall, 3 major themes emerged from the discussion thread: 1 emphasizing the importance of intentional control, 1 highlighting the unavoidable prominence of tinnitus, and 1 drawing attention to the resulting cognitive effort and mental fatigue required when concentrating.

#### Intentional Control

One emerging theme was the importance of being able to control your *concentration,* including the ability to focus on whatever you wish or need to focus on at any given time and the ability to control the switching of attention. One discussant said: “So concentration is the ability to exercise attentional control, to stay focused on something of our own choice. This ability is impeded when you have tinnitus.”

#### Centrality of Tinnitus

Discussion of *concentration* demonstrated the unavoidable prominence of tinnitus. Discussants struggled to suggest plain language definitions for the outcome domain that did not include the word tinnitus, despite specifying that this was undesirable as it gave more power or contradicted the notion of *concentration* being the ability to focus elsewhere and on other things. For example, 1 discussant wrote “I feel with the wording ‘away from the tinnitus’, just by virtue of that wording and sentiment implies you are trying so hard to distract yourself that the tinnitus remains the focus.”

#### Effortfulness

The concept of *concentration* was agreed to encompass not only the ability to focus and function cognitively, but also the additional mental effort required because of the presence of tinnitus and the subsequent fatigue caused by that effort. One discussant said:

I would consider a sound-based treatment successful if it restored, even partially, my ability to immerse myself in a task and for this to feel less of an effort than it is now. Ideally, the treatment should reduce the occurrence of cognitive tiredness that makes sustained concentration difficult.

To conclude, the revised definition of *concentration* was as follows: “The ability to keep your attention focused on whatever you wish.” This was described by discussants to be referring to the ability to control your attention and sustain focus on whatever it is you intend to focus upon, with successful tinnitus interventions enhancing the ability to concentrate by making it easier and less effortful.

#### Ability to Ignore

Overall, 2 major themes emerged from the discussion thread: one debating whether the *ability to ignore* should be attributed to the tinnitus sound itself or to individual capabilities and another raising concerns about the negative connotations of the choice of wording, including whether or not ignoring tinnitus represents a realistic goal.

#### Changes in the Tinnitus Percept, Not Just in the Individual’s Capabilities to Ignore

A key debate focused on whether the concept referred to any change in the tinnitus percept (ie, making the noise easier to ignore) or in the individual’s capability (ie, making the person better at ignoring tinnitus). Some discussants expressed no strong preference: “Either outcome/effect (change in tinnitus or in person) would be good.” However other discussants agreed that a reasonable expectation for sound-based treatments, the only intervention type that this outcome domain is recommended for, is to improve *ability to ignore* by a third mechanism that falls somewhere in between, “a sound-based treatment can’t really affect your personal abilities (in the same way as a psychological treatment might) but nor can it change the tinnitus itself (from my experience). To give an analogy, glasses don’t change my eyesight or improve my ability to be observant—they just allow me to see better whilst I am wearing them.” As it was not unanimously resolved, this theme did not lead to any change to the plain language definition of *ability to ignore.* However, it was still a substantial theme that emerged from the discussion and may be informative in next steps deciding how the *ability to ignore* should be measured, as what sort of questions should be asked is best governed by what sort of change a successful intervention is likely to create and what sort of change would be meaningful to patients.

#### Negative Connotations and Unrealistic Goals

Concerns were voiced about negative connotations of the word *ignore* and discussants were worried that it could be seen as dismissive toward tinnitus or as blaming people living with tinnitus for their own suffering. As 1 discussant reasoned: “Replace tinnitus with any other disease: as if cancer were not there, depression were not there... and suddenly it makes no sense at all.” There were also criticisms of the original definition of this outcome domain as an unrealistic or unachievable goal, and for its use of the term *normal*: “What is normal? We all change and adapt to what life throws at us.”

To conclude, discussants seemed most supportive of an understanding of the *ability to ignore* as adapting to tinnitus, adjusting daily life and routine activities so as to minimize the negative impact of tinnitus, and maintaining healthy and realistic goals for how a treatment may be able to help make tinnitus easier to ignore in certain situations or to certain extents. The revised definition was as follows: “The ability to continue as if tinnitus were not there.”

## Discussion

### Principal Findings

The 5 Web discussion forums brought together a self-selected subset of survey participants, including health care users and professionals with experience of tinnitus. Participants took part in semistructured discussions of 5 complex concepts relating to patient-reported tinnitus-specific complaints that had been voted during a preceding e-survey. The qualitative data collected during these discussion threads provide an important in-depth understanding of each health-related concept, which had not been possible hitherto. The descriptive summaries and revised definitions also provide clarification on aspects of similarity and distinctiveness between core outcome domains. These findings are informative for identifying outcome instruments that putatively assess these concepts and for evaluating their content validity [6,13,35].

Despite the paucity of qualitative data from people with tinnitus [1], 1 study using interviews to explore preferences for outcomes and treatments confirms 3 of the present construct descriptions [36]. In response to a question about what they were hoping for in a treatment, patients described their preference for a “reduction of conscious awareness of the tinnitus (to reduce time listening to tinnitus).” This notion reflects a theme from the Web forum discussion on the *ability to ignore*. Preferences for how they would like to receive treatments highlighted the importance of “choice in personalising their care and determining the best course of action for them,” which is synonymous with our interpretation of the construct *sense of control.* Psychological adjustment (described by 1 participant as “you’ve got to learn to accept it”) was understood to be an active part of coping with tinnitus in a similar way that we have described the construct *acceptance of tinnitus.* Although the study by Pryce et al [36] was exploratory and only interviewed 41 patients based in the United Kingdom, it is nevertheless important because it presents an independent look at similar issues and was unpublished at the time this study was ongoing.

### Strengths of Using a Web-Based Peer Discussion Forum for Core Outcome Set Development

An increasing number of social and health science researchers are recognizing the internet as a rich source of information. A Web discussion forum facilitates participation by any number of individuals in a way that is not constrained by geographical location or time zone. It offers a rapid and easy way to engage with a large number of participants whilst being more flexible and cost-effective than conventional face-to-face methods. Furthermore, the data are already transcribed, and so they are less likely to contain errors and are immediately ready to analyze (see the study by Ferrante et al [37] for a review). Unsurprisingly, an increasing number of investigators are designing Web discussion forums to collect qualitative research data from patients and using thematic analysis to evaluate forum posts [38-40]. This study contributes to the increasing use of electronic communication to support group decision making and consensus making [41].

A major strength of using such a virtual environment is that it allows investigators to conduct real-time qualitative analyses as part of an iterative process in which the participants are actively involved in determining the meaning and significance of findings and where the moderators are able to consolidate, clarify, and resolve any misunderstandings for the purposes of concept definition. The Web format seemed to provide a suitable space that enabled participants to reflect and share ideas about word choice and semantics. With the exception of *concentration*, all discussion threads had a rich debate on choice between alternative wordings. For example, 1 discussant said: “Clearly the same words can mean or imply very different things to different people. That’s inevitably going to be a big issue with tinnitus which is so individual to each person affected by it.” Written forum posts, perhaps more so than spoken conversations, lend themselves to greater deliberation over the selection of a particular word according to how it might be interpreted. Discussion about the words *acceptance* and *ignore* are good examples. As 1 discussant said:

Imagine not being part of this forum, but being told by your doctor that some new treatment leads to your being able to ignore the tinnitus, only to find out that it doesn’t for you, and then only worked in the research because the definition was engineered in a particular way... I suppose what I’m saying is that ability to ignore (and the other definitions) have to reflect what most tinnitus sufferers would think if they heard that phrase from their doctor or therapist.

In this way, discussants were not just research participants, they also played an important role in shaping the research *product*. The Web discussion forum confirms the usefulness of each 2-week discussion period, to consolidate, clarify, and resolve any misunderstandings.

One of the positive themes emerging from the Web forum discussion was the therapeutic benefit of participation. Several discussants living with tinnitus thanked the moderator and research team, expressing a sense of reward in having taken part and describing how it had been personally enlightening and therapeutic. One said: “This is better than I felt 2 years ago, and I must admit that this academic research group with fellow sufferers has been a part of that improvement.” Another said: “Lastly I just wanted to say thanks to ’Manager’ and to COMIT'ID and everyone else for all of this. It’s been really good/therapeutic for me to have been a part of it, and to be able to hear all the experiences of everyone else.” Although Web discussion forums are commonplace for peer support groups [42], similar benefits through sharing ideas and experiences during a research study should not be overlooked. It was not necessary for the moderator to offer support, as the participants took on that role themselves (see also the study by Ferrante et al [37]). We feel it is important to acknowledge that this happened despite the identity of health care users or professionals not being known. This may have helped to create a space without hierarchies where interpersonal relationships were on a level playing field. For example, 1 discussant responded to another:

I don’t think anyone is nit-picking. The purpose of these discussions is to better understand what the core outcomes mean to everyone and reach a better agreement about their definitions. Everyone’s views are valid, equally important to hear and worthy of respect. The whole point of the research is to achieve as broad a consensus as possible and this can only be achieved by exploring and discussing where and how our views align and where they differ.

A mix of health care users and professionals should also help to avoid some of the potential for bias in the design and interpretation of the study if carried out only by a particular stakeholder group (eg, health care users) [5].

### Limitations of This Web-Based Peer Discussion Forum for Core Outcome Set Development

A potential limitation of using Web forums for data collection is that participants need to be computer literate and able to communicate adequately in written English. This may limit the population somewhat and bias self-selection toward those who are more health literate. For this particular study design, it is possible that opening and closing discussion threads in sequence could have resulted in participants who joined later on in the 6-week process missing the opportunity to share their viewpoints on earlier outcome domains. It is also possible that within a different context or procedure, more themes could emerge as it was not possible to ascertain whether data saturation was reached by the forum discussions [43,44]. However, the main practical study objective was to enable robust decision making for the 5 core outcome domains in a time-limited way. We believe that this was successfully achieved at least for the sense of control, acceptance of tinnitus, concentration, and ability to ignore. In the case of tinnitus intrusiveness, the Web discussion forum was perhaps more limited in its ability to converge opinions onto a plain language definition as the concept appears to be particularly complex and viewpoints are more variable.

### Implications for Future Research

#### Expanding the Subdomains Encompassed by the Concept Tinnitus Intrusiveness

During preparation for the electronic Delphi survey, the COMiT team, with input from health care users, had made a decision to narrowly define the outcome domains, removing broad concepts that were reflected in a number of more narrowly focused outcome domains [9]. However, during the face-to-face consensus meetings and these Web discussion forums, participants argued for a different approach, noticing where concepts were interrelated and favoring to nest those interrelated concepts under a broader construct definition. For example, health-related quality of life was originally deemed to be a broad concept encompassing subdomains such as *impact on relationships, impact on individual activities*, *impact on social life*, and *impact on work* [9]. We had considered these subdomains as distinct outcome domains in their own right, but the overwhelming opinion of stakeholders was that they should be incorporated into the construct *tinnitus intrusiveness* [11]. The next challenge will be to examine the spectrum of symptoms and aspects of functioning and health that health care users, health care practitioners, clinical researchers, and other stakeholders, such as regulatory agencies, expect to be covered in a measure of *tinnitus intrusiveness*. Precedents for this next step exist in other disciplines, such as deciding how to measure quality of life in chronic pain [16] and quality of life in adults with eczema [45].

#### Evaluating the Content Validity of Existing Instruments

Content validity refers to a number of key attributes of a measurement instrument, namely, how relevant the items are for the construct and target population of interest, how comprehensively those items reflect the construct, how comprehensible the instrument is, and whether it is understood by patients as intended [35]. Content validity is often considered to be the most important measurement property of a patient-reported outcome measure because its lack can undermine all other measurement properties. The rich personal insights reported in this study provide a firm foundation for defining exactly what symptoms and aspects of functioning that health care users might expect to be covered in the measurement tools that assess the 5 concepts of interest. One start would be to use the themes emerging from this study to create a thematic *checklist* that can then be compared against the item content of available instruments [45], while evaluating the adequacy of the published evidence for their content validity [13,35]. To assist this process, the in-depth executive summaries and proposed subdomains can be taken alongside the plain language definitions. We further suggest that this approach could be applied to outcome domains where the Web discussion forum did not reach an agreement on the concept definition (such as *tinnitus intrusiveness*) and outcome domains that are more typically measured using performance-based tests rather than questionnaires (such as *concentration*).

### Conclusions

Our experience leads us to strongly advocate the use of qualitative methods to ensure concepts are defined to support clear and consistent interpretation by all end users and agreed upon before looking to map outcome domains to measurement instruments. The vast range of different interpretations held for the same domains became apparent during the study, and some major decisions were made as to how the core outcome domains should be conceptualized, defined, and distinguished going forward. Any COS development study following the recommendations of COMET [5] should place substantial emphasis on patient and public involvement. This necessitates involving stakeholders in detailed concept definition as it cannot be assumed that those who contribute to the consensus decision are all speaking the same language based upon research literature and professional terminology. Our findings support the acceptability and feasibility of using Web discussion forums as a research method to achieve this.

## Appendix

Multimedia Appendix 1 Supplementary analysis of consensus meeting discussion on the 4 core outcome domains that were not part of the Web discussion forum.

Multimedia Appendix 2 Instructional Video 1. Hosted on the homepage, walking participants through how to register and create an account.

Multimedia Appendix 3 Instructional Video 2. Hosted on the homepage once logged in, walking participants through how to post, comment on others posts, and adjust their notification settings.

Multimedia Appendix 4 Moderator’s semistructured plans for each discussion thread, including general template of prompts, questions, and posts and tailored discussion packs for each core outcome domain.

Multimedia Appendix 5 Executive summaries and revised plain language definitions for each core outcome domain, including voting results for each discussion thread.

## Abbreviations

COMET: Core Outcome Measures in Effectiveness Trials
COMiT: Core Outcomes Measures in Tinnitus
COMiT’ID: Core Outcomes Measures in Tinnitus: International Delphi
COS: core outcome set
NIHR: National Institute for Health Research
