# Supplementary material for: Defining Symptom Concepts in Chronic Subjective Tinnitus: Web-Based Discussion Forum Study
Source: Interact J Med Res. 2020 Jan 7;9(1):e14446. doi: 10.2196/14446 (PMC6996772; doi:10.2196/14446)
Supplement: Multimedia Appendix 1 [file ijmr_v9i1e14446_app1.docx]

# Progressing towards a common standard: using an online discussion forum to define concepts in chronic subjective tinnitus symptomatology

# Multimedia Appendix 1: Supplementary analysis of consensus meeting discussion on the four core outcome domains which were not part of the online discussion forum.

## Aim

To consolidate and finalise the four remaining core outcome domains not discussed in the moderated online discussion forum (*quality of sleep*, *mood*, *negative thoughts and beliefs*, and *tinnitus loudness*). This includes reporting the agreed plain-language definition, identifying where the definition needed expanding to capture other outcome domain concepts (as subdomains), and gaining a more in-depth understanding of each concept. This is to bring all of the nine core outcome domains to the same point, in preparation for the second step in the Core Outcome Set (COS) development which is to determine *how* those outcomes should be measured.

## Method

Thematic analysis of transcripts of three one-day face-to-face consensus meetings and two half-day patient and public involvement (PPI) meetings which had taken place earlier in the COMiT’ID study [1, 2].

Transcription and thematic analysis focused on any mention of the four core outcome domains of interest: *quality of sleep*, *mood*, *negative thoughts and beliefs*, and *tinnitus loudness*.

Methods for thematic analysis followed Braun and Clarke’s six-stage framework [3], and qualitative analysis was conducted using QSR International’s NVivo Pro 11 software (NVivo qualitative data analysis Software; QSR International Pty Ltd. Version 11, 2015) by one analyst (AH). The text was read several times for familiarisation, and then it was coded. Themes arising from the conversation were identified by grouping codes, and then were refined and defined through an iterative process. Coded text segments provided the evidence corresponding to each theme. For these concepts, there was no input from a second analyst nor the Principal Investigator.

## Results

The following table describes the original plain language definitions and the expansions to each concept with respect to which subdomains should be included in the operational definition, as recommended by participants in the consensus meetings.

| **Outcome domain** | **Original plain language definition** | **Subdomains** |
| --- | --- | --- |
| *Quality of sleep* | Getting the right amount of undisturbed sleep for you that leaves you feeling refreshed and rested | Difficulties getting to sleep (maybe) |
| *Mood* | A general sense of well-being, ranging from feeling very low or negative to very positive | Depressive symptoms  Anxiety |
| *Negative thoughts and beliefs* | Thinking tinnitus will affect you in a negative way (e.g. thinking that tinnitus is never going to get better or that It would be dreadful if these noises never went away) |  |
| *Tinnitus loudness* | How loud your tinnitus sounds |  |

## In-depth understanding of each concept

The themes under each core outcome domain are not reported in any particular order.

### Quality of sleep

#### Management strategies

A major theme that emerged during discussion of *quality of sleep* was the diverse range of strategies that tinnitus experts discover, develop and employ to enhance quality of sleep. Some of those mentioned include recommended or prescribed sound interventions, such as “cochlear implants”, “hearing aids” or “a digital sound pillow”, whereas others were everyday items and techniques found to be helpful. For example “the radio”, “listening to an audio book”, “a sleep app machine” or simply as a couple of discussants agreed: “I don’t stop all day…so that when I got to bed, I’m exhausted, so I do sleep straight away”. It is important that any measure of *quality of sleep* used in a tinnitus clinical trial recognises the presence of different individual strategies to ensure purity that any improvements definitely result from the intervention being assessed, as well as the different ways people may continue to struggle with *quality of sleep* even if certain aspects of sleep seem to improve. For example, several discussants shared personal experiences of tackling how to get to sleep, but then being woken by the tinnitus throughout the night: “when I take my hearing aid out last thing at night, for about half an hour… I’ve actually been able to get to sleep without the roaring tinnitus. And then it comes back at 4 in the morning when my brain is supposed to be asleep, it just kicks in again”

#### Fundamental determinant of quality of life

Despite *quality of sleep* being selected as a core outcome domain for assessing the efficacy of sound-based tinnitus interventions, some discussants argued that *quality of sleep* was not so critical because not everyone with tinnitus experiences problems sleeping. However, it was reasoned that sleep is still the most common complaint cited within tinnitus literature, and that the impact of poor sleep is so great, both for everyday functioning: “if you don’t get the quality of sleep, you start to have I’m tired the next day, I can’t concentrate on my work, I can’t focus on driving the car”, and for emotional and psychological wellbeing: “sleep is the only way that you can switch off, psychologically, mentally, physically. If you don’t switch off with sleep…then you’re in a vicious spiral, and a downward spiral. And that’s where all your depression and your anxiety and the rest start coming up to the fore.”

#### Collapsing two domains together

Many attendees at the sound-based interventions consensus meeting found it difficult to distinguish between *quality of sleep* and a second domain in consideration, *difficulties getting to sleep*. Recognising the importance of both domains, and not wanting to lose aspects of either, several attendees made similar suggestions: “I would have thought they could be put together”. However this was not taken to a vote. *Quality of sleep* was selected for the core outcome domain set, while *difficulties getting to sleep*  was set aside. It is possible that *quality of sleep*  is the higher-level domain: “if you’re not getting to sleep then you’re not having quality sleep are you? So it should be included in the overall quality of sleep I’d have thought”. It is also possible that *difficulties getting to sleep* may or may not in practice be measured as a sub-domain of *quality of* sleep depending upon future stages of the COMiT initiative identifying measurement instruments for each core outcome domain. As one consensus meeting attendee alluded: “From a clinician point of view, pretty much everyone gets the tinnitus functional index now and there’s 3 questions on sleep in there, and I always think it’s far too much. You just want to know: do you have difficulties sleeping? Whether it’s getting to sleep or whether its waking up again.”

### Mood

#### Spectrum

*Mood* was defined as “an overarching term”, with emphasis placed on how it is best understood as a “continuum” or spectrum “ranging from feeling very low or negative to very positive”, covering the full valence and variety of emotions imaginable. For example, using anxiety as an example, it was finally agreed that *mood* includes low-level “feelings of being anxious as a part of the everyday human condition” all the way up to feelings “so severe that a clinician would say you have a generalised anxiety problem”.

#### Expansion to include sub-domains

During discussion of *mood* at the psychology-based interventions consensus meeting, many attendees highlighted its similarity to other domains *anxiety* and *depressive symptoms* in their attempt to prioritise and select just one: “If we had an agreed definition that mood does capture both then I think we don’t need both.” It was taken to a vote and agreed by 100% of attendees that mood should explicitly include both *anxiety* and *depressive symptoms* as sub-domains. As one attendee reasoned: “I think what we need to ask ourselves is if the treatment that we’re investigating makes people less anxious or less depressed, is that going to be reflected in a measure of overall mood? And I think it probably is.”

#### General sense of wellbeing

*Mood* was defined throughout discussions as an individual’s “general state of wellbeing”. This seems to be referring to one’s “average everyday mood” or “overall state of mind” and importantly “how it can be changed by [their] tinnitus”. Some participants seemed to identify *mood* as a place to measure any and all of the potential “emotional reaction” to tinnitus in daily life, from “despondency” to “agitation”. However as one participant said “a lot of people are glass half full and a lot of people are glass half empty” emphasising the importance of taking into account an individual’s general baseline mood state to accurately measure any improvements made by ameliorating the impact of the tinnitus.

### Negative thoughts and beliefs

#### Pessimism and catastrophising

This core outcome domain refers to *negative thoughts and beliefs* about tinnitus such as “that they’ve got a brain tumour or something of that nature”, “that their life is going to be shortened” or that the “tinnitus is never going to get better”. It was clarified as of key importance when those thoughts are irrational, remain completely unbalanced by any positive thoughts or are left unevaluated and corrected by the individual: “I mean everybody will have negative thoughts sometimes won’t they? But some people will just dismiss them and other people will dwell on them and live by them.” Discussions made clear that this domain must separate the “pessimistic, defeatist, gloomy, cynical, bleak” thoughts from the overall outlook or associated emotions, with this domain intended to reflect just the thoughts and beliefs themselves.

#### Demonstration of the tinnitus journey

A key theme that emerged during discussion of the domain *negative thoughts and beliefs* was how it reflects people’s journey with tinnitus. Discussants shared examples indicative of missing the past and their life before tinnitus, and hope, or lack thereof, for their future with tinnitus: “well when I first started with tinnitus my overwhelming problem was negative thoughts around – and in fact when I saw that I thought that explains exactly what my problem was, was that I had all these negative thoughts about tinnitus, about how it’s going to affect me and the rest of my life, you know, my social life, my work life, everything.” This quotation also demonstrates a recurring theme that the early days of having tinnitus are characterised by this domain: “it’s your first - those scary thoughts that everybody has”, and that this may shows improvement as the journey progresses and with the help of psychology-based interventions. The same individual went on to say: “And the difference between then before and after I’ve had psychological treatment, care, was that those negative thoughts had gone away. My tinnitus is still intrusive. It’s always intrusive; one day it’s not, one day it is. But I’ve stopped having the negative thoughts.” It was also highlighted that individuals may not “own up to” having extreme, perhaps irrational, n*egative thoughts and beliefs* whilst in the midst of suffering from them, but as they overcome them they are more able to recognise them as such and relate to this domain.

### Tinnitus loudness

#### A direct measure of tinnitus

This domain was supported as being the closest thing to a direct measure of tinnitus itself, in contrast to the other domains, which tap into secondary effects and impacts of the tinnitus. While *tinnitus loudness* would most likely still be based on subjective patient-reported measurement, some felt that it is the most simple and, in some ways, “objective” quantitative measure of tinnitus as it should not “depend enormously on the personality of the person”. It was also argued that this domain is essential in order to “evaluate what is a clinical meaningful change”; although a more simple approach assumes “eliminate the noise, eliminate the intrusion”, measuring *tinnitus loudness* alongside the other core outcome domains allows validation of any change resulting from a treatment: “How much do you affect the phenomenon, and how much does that effect benefit the patient.”

#### Beyond just the volume

While at no point voted upon or officially confirmed as being a consensus opinion, it was suggested and implied at several points in development and consensus meeting discussions that this domain should perhaps refer to all aspects of the tinnitus sound. This may include “the intensity”, “the quality of the sound”, “the pitch”, “the sensation”, the “unpleasantness” and any other acoustical or audiometric element of the tinnitus.

## Acknowledgements

We thank Dr. Sarah Michiels, Dr. Padraig Kitterick, and Dr. Derek Hoare for facilitating the consensus meeting discussions. Participants in the consensus meetings were as follows: sound-based interventions (Remo Arts, Michelle Booth, Peter Byrom, Philippe Fournier, Anna Frost, Claire Gatenby, Michael Golenhofen, Saskia Harden, Steve Harrison, Derek Hoare, Iain MacLeod Brudenell, Ian McCluskey, Jane McFerran, Robert Pierzycki, Penny Peake, Stephanie Polak (Fuller), Margaret Shelton, Rosemary Shippard, Gail Webb); psychology-based interventions (Martin Clarke, Nicolas Dauman, Gwenda Eckersley, Lucy Handscomb, Alan Hopkirk, Laure Jacquemin, Max Millar, Noel Plummer, Helen Pryce, Zofia Pucek, Robert Rainford, Sylvia Roberts, Charlotte Rogers, Jacqui Sheldrake, Jeremy Thomas, Dean Thompson, Barbara White, Darren Wild, Tadeusz Woroniecki); and pharmacology-based interventions (Sandra Bastos, James Blackwell, Benjamin Böcking, Mark Day, Juan Domènech, Jonas Dyhrfjeld-Johnsen, Ralph Holme, Tobias Kleinjung, John Rowley, Carina Santos, Richard Southcott, Thomas Suender, Agnieszka Szczepek, Nuno Trigueiros-Cunha, Paul Van de Heyning, Silvia Zaragoza-Domingo).

## References

1. Fackrell K, Smith H, Colley V, Thacker B, Horobin A, Haider HF, Londero A, Mazurek B, Hall DA. Core Outcome Domains for early phase clinical trials of sound-, psychology-, and pharmacology-based interventions to manage chronic subjective tinnitus in adults: the COMIT'ID study protocol for using a Delphi process and face-to-face meetings to establish consensus. Trials 2017 Aug; 18(1): 388. PMID: 28835261

2. Hall DA, Smith H, Hibbert A, Colley V, Haider HF, Horobin A, Londero A, Mazurek B, Thacker B, Fackrell K, for the Core Outcome Measures in Tinnitus (COMiT) initiative. The COMiT’ID study: developing core outcome domains sets for clinical trials of sound-, psychology-, and pharmacology-based interventions for chronic subjective tinnitus in adults. Trends Hear 2018 Jan-Dec; 22: 1-16. PMID: 30488765

3. Braun V, Clarke V. Chapter 4: Thematic analysis. In: Cooper H, Camic PM, Long DL, Panter AT, Rindskopf D, Sher KJ, editors. APA Handbook of research methods in psychology: Vol. 2. Research Designs: Quantitative, Qualitative, Neuropsychological, and Biological. Washington, DC: American Psychological Association; 2012. p. 57-71. ISBN: 978-1-4338-1003-9
