# Supplementary material for: Defining Symptom Concepts in Chronic Subjective Tinnitus: Web-Based Discussion Forum Study
Source: Interact J Med Res. 2020 Jan 7;9(1):e14446. doi: 10.2196/14446 (PMC6996772; doi:10.2196/14446)
Supplement: Multimedia Appendix 4 [file ijmr_v9i1e14446_app4.docx]

# Progressing towards a common standard: using an online discussion forum to define concepts in chronic subjective tinnitus symptomatology

# Multimedia Appendix 4: Moderator’s semi-structured plans for each discussion thread including general template of prompts, questions and posts, and tailored discussion packs for each core outcome domain.

## Template of prompts, questions and posts

**At open:**

The current definition of x is ……

What do you like about this definition and what do you think could be changed or improved?

**Week 1:**

What does x mean to you?

Remembering that x has been chosen as a core outcome for [type of intervention], what aspects of x would you expect a successful [type of] intervention to change?

(choose best phrasing:) What experiences and aspects of tinnitus would you want a questionnaire about x to ask about? Is there anything that, if it wasn’t mentioned, would make you feel like the questionnaire had failed and missed the point of x? / What experiences and aspects of x would you want a questionnaire about tinnitus to ask about? Is there anything that, if it wasn’t mentioned, would make you feel like the questionnaire had failed and missed the point of x with tinnitus?

**Week 2:**

At the consensus meetings, it was discussed that x should include/cover/reflect a b c. What do you think about that? Do we think it belongs within the definition of x?

...It was also discussed that a b c may fit within the other core outcome(s) of y or z. Where do you think it fits best?

Have definitions for y and z on hand

**Approx. 4 days before close:**

Thank you so much for all of your discussion, it has been really useful having your ideas and opinions to guide us in this difficult process of defining the core outcomes, and we appreciate the time you have dedicated to taking part. To summarise, it seems/we have decided/it seems we all agree ….(things decided to be added that the outcome should cover, changes to/issues with the original definition, any remaining debates or disagreements if definitely unresolvable). Therefore, a new definition for the outcome x may be…..

Poll: Do you agree with the summary and new definition?

1. Yes, it accurately summarises the discussion and I agree with the new definition
2. It accurately summarises the discussion but I do not agree with the new definition
3. No, it does not accurately summarise the discussion and I do not agree with the new definition

**At close:**

Thank you all for taking part. This discussion is now closed. If you were unable to take part while the discussion was open for any reason, or have any final comments that you want to add, you can send them to [hearing NIHR email address?]. We will not be able to change any of the discussion’s conclusions or changes made to the definition but we may use late comments to inform our decisions in the next stage of the research, and they may be included in the reporting of these discussions.

**Note: Add questions asked so far into first message as an edit**

## Tinnitus intrusiveness discussion pack

The original definition of tinnitus intrusiveness is “Noticing the sound of tinnitus is there and it is invading your life or your personal space”.

What do you like about this definition and what do you think could be changed or improved?

What does tinnitus intrusiveness mean to you?

Remembering that tinnitus intrusiveness has been chosen as a core outcome for all three types of tinnitus intervention, what aspects of tinnitus intrusiveness would you expect a successful sound, psychology or pharmaceutical intervention to change?

What experiences and aspects of tinnitus would you want a questionnaire about intrusiveness to ask about? Is there anything that, if it wasn’t mentioned, would make you feel like the questionnaire had failed and missed the point of tinnitus intrusiveness?

OR

What experiences and aspects of intrusiveness would you want a questionnaire about tinnitus to ask about? Is there anything that, if it wasn’t mentioned, would make you feel like the questionnaire had failed and missed the point of intrusiveness with tinnitus?

At the consensus meetings, it was discussed that some people felt intrusiveness should reflect the extent to which tinnitus is preventing general holistic health, emotional wellbeing, and daily life. What do you think about that? Is that what the definition of tinnitus intrusiveness should be, or is that too much?

At the consensus meetings, it was discussed that some people felt intrusiveness should reflect general quality of life with tinnitus. What do you think about that? Is that what the definition of tinnitus intrusiveness should be, or is that too much?

At the consensus meetings, it was discussed that tinnitus intrusiveness should include all of the impact domains (impact on work, impact on relationships, impact on social life, and impact on individual activities). What do you think about that? Do we think they belong within the definition of intrusiveness?

It was also discussed that the impact domains may fit within the other core outcomes of concentation and ability to ignore. Where do you think they fit best?

*Definitions:*

*Impact on individual activities: Effect of tinnitus on your choice to engage in your individual interests or tasks (e.g. driving, reading, listening to music or watching TV). Not group activities*

*Impact on relationships: Effect of tinnitus on relationships with family and friends*

*Impact on social life: Effect of tinnitus on the ability to take part fully in a group social gathering (e.g. at a restaurant, at the park or at a party)*

*Impact on work: Effect of tinnitus on your ability to carry out work tasks or job roles*

At the consensus meetings, it was discussed that tinnitus intrusiveness should include annoyance and irritability. What do you think about that? Do we think they both belong within the definition of intrusiveness?

It was also discussed that annoyance and irritability may fit within the other core outcomes of acceptance of tinnitus, ability to ignore, or mood. Where do you think they fit best?

OR

At the consensus meetings, it was discussed that tinnitus intrusiveness should include annoyance. What do you think about that? Do we think that belongs within the definition of intrusiveness?

It was also discussed that annoyance may fit within the other core outcomes of acceptance of tinnitus, ability to ignore, or mood. Where do you think it fits best?

At the consensus meetings, it was discussed that tinnitus intrusiveness should include irritability. What do you think about that? Do we think that belongs within the definition of intrusiveness?

It was also discussed that irritability may fit within the other core outcomes of acceptance of tinnitus, ability to ignore, or mood. Where do you think it fits best?

*Definitions:*

*Annoyance: Noticing the sound of tinnitus is there and it feels like a nuisance*

*Irritability: Having a tendency to easily feel tense, on edge or agitated because of your tinnitus*

At the consensus meetings, it was discussed that tinnitus intrusiveness should include tinnitus awareness and tinnitus unpleasantness. What do you think about that? Do we think they both belong within the definition of intrusiveness?

It was also discussed that tinnitus awareness and tinnitus unpleasantness may fit within the other core outcome of tinnitus loudness. Where do you think they fit best?

OR

At the consensus meetings, it was discussed that tinnitus intrusiveness should include tinnitus awareness. What do you think about that? Do we think that belongs within the definition of intrusiveness?

It was also discussed that tinnitus awareness may fit within the other core outcome of tinnitus loudness. Where do you think it fits best?

At the consensus meetings, it was discussed that tinnitus intrusiveness should include tinnitus unpleasantness. What do you think about that? Do we think that belongs within the definition of intrusiveness?

It was also discussed that tinnitus unpleasantness may fit within the other core outcome of tinnitus loudness. Where do you think it fits best?

*Definitions:*

*Tinnitus awareness: Noticing the sound of tinnitus is there*

*Tinnitus unpleasantness: Tinnitus making you feel disagreeable or uncomfortable*

At the consensus meetings, it was discussed that tinnitus intrusiveness should include coping. What do you think about that? Do we think that belongs within the definition of intrusiveness?

It was also discussed that coping may fit within the other core outcomes of acceptance of tinnitus or sense of control. Where do you think it fits best?

*Definition:*

*Coping: Ability to deal with or handle tinnitus*

Thank you so much for all of your discussion, it has been really useful having your ideas and opinions to guide us in this difficult process of defining the core outcomes, and we appreciate the time you have dedicated to taking part. To summarise, it seems/we have decided/it seems we all agree [things decided to be added that the outcome should cover, changes to/issues with the original definition, any remaining debates or disagreements if definitely unresolvable]. Therefore, a new definition for the outcome acceptance of tinnitus may be […]

Poll: Do you agree with the summary and new definition?

1. Yes, it accurately summarises the discussion and I agree with the new definition
2. It accurately summarises the discussion but I do not agree with the new definition
3. No, it does not accurately summarise the discussion and I do not agree with the new definition

Thank you all for taking part. This discussion is now closed. If you were unable to take part while the discussion was open for any reason, or have any final comments that you want to add, you can send them to [hearing NIHR email address?]. We will not be able to change any of the discussion’s conclusions or changes made to the definition but we may use late comments to inform our decisions in the next stage of the research, and they may be included in the reporting of these discussions.

## Sense of control discussion pack

The original definition of sense of control is “Whether or not you feel you have a choice in how to manage the impact of tinnitus and feelings caused by tinnitus”.

What do you like about this definition and what do you think could be changed or improved?

What does sense of control mean to you? What experiences and aspects of sense of control with tinnitus would you want a questionnaire to ask about? Is there anything that, if it wasn’t mentioned, would make you feel like the questionnaire had failed and missed the point?

Remembering that sense of control has been chosen as a core outcome for psychology-based and sound-based interventions for tinnitus, what aspects of sense of control would you expect a successful sound and/or psychology –based intervention to change?

At the consensus meetings, it was discussed that some people felt sense of control should reflect a sense of control over the tinnitus itself, while others felt it should reflect a sense of control over the symptoms of the tinnitus, their own feelings and reactions towards the tinnitus and the impact is has on themselves and their lives. What do you think?

At the consensus meetings, it was discussed that possibly the definition for sense of control, and the expectation for change to sense of control, may be different for sound interventions and psychology interventions e.g. a good sound intervention may make it easier to control the tinnitus itself, while a good psychology intervention may make it easier to manage their own reaction to the tinnitus and how much it negatively affects their wellbeing and life. What do you think about this?

OPTIONAL: Considering the aim is to have one definition and ultimately one measure for each outcome domain, how do you think sense of control should be defined for both sound and psychology-based interventions? Do you have any ideas how this distinction between the two could be brought back together?

At the consensus meetings, it was discussed that sense of control should be considered the opposite to the domain helplessness (lack of control). What do you think about that? Do we think that belongs within the definition of intrusiveness?

Additional question about how that means sense of control is not necessarily becoming able to control their tinnitus, but letting go of that need to control it and therefore overcoming/getting rid of the sense of despair that results from a struggle for control???

*Definition:*

*Helplessness (lack of control): Feeling despair about being unable to control or manage tinnitus*

At the consensus meetings, it was discussed that some people felt sense of control should encompass self-efficacy, which is defined as “an individual’s belief in their ability to succeed in a specific situation or accomplish a task”, and some people felt sense of control should encompass self-confidence. What do you think about that? Are these concepts relevant and similar to the outcome domain of sense of control?

OPTIONAL: How do you feel "sense of control" is distinct from "acceptance"? In what ways are they different and how can we pick them apart?

OPTIONAL: At the consensus meetings, it was discussed that some people did not like the word “control” as they know that they cannot control their tinnitus, while others felt that it was the correct and best word for the definition and the sense of not being helpless or powerless to their tinnitus symptoms. What do you think about that? Is the word “control” the right term for the outcome or would you prefer it to have a different name?

Additional question – “coping” fits here or in acceptance? (what were the results of the discussion in acceptance?)

Thank you so much for all of your discussion, it has been really useful having your ideas and opinions to guide us in this difficult process of defining the core outcomes, and we appreciate the time you have dedicated to taking part. To summarise, it seems/we have decided/it seems we all agree [things decided to be added that the outcome should cover, changes to/issues with the original definition, any remaining debates or disagreements if definitely unresolvable]. Therefore, a new definition for the outcome acceptance of tinnitus may be […]

Poll: Do you agree with the summary and new definition?

1. Yes, it accurately summarises the discussion and I agree with the new definition

2. It accurately summarises the discussion but I do not agree with the new definition

3. No, it does not accurately summarise the discussion and I do not agree with the new definition

Thank you all for taking part. This discussion is now closed. If you were unable to take part while the discussion was open for any reason, or have any final comments that you want to add, you can send them to [hearing NIHR email address?]. We will not be able to change any of the discussion’s conclusions or changes made to the definition but we may use late comments to inform our decisions in the next stage of the research, and they may be included in the reporting of these discussions.

## Acceptance of tinnitus discussion pack

The current definition of acceptance of tinnitus is “Recognising that tinnitus is a part of your life without having a negative reaction to it”.

What do you like about this definition and what do you think could be changed or improved?

What does acceptance of tinnitus mean to you?

Remembering that acceptance has been chosen as a core outcome for psychology-based interventions for tinnitus, what aspects of tinnitus acceptance would you expect a successful sound-based intervention to change?

What experiences and aspects of tinnitus would you want a questionnaire about acceptance to ask about? Is there anything that, if it wasn’t mentioned, would make you feel like the questionnaire had failed and missed the point of tinnitus acceptance?

OR

What experiences and aspects of acceptance would you want a questionnaire about tinnitus to ask about? Is there anything that, if it wasn’t mentioned, would make you feel like the questionnaire had failed and missed the point of acceptance with tinnitus?

At the consensus meetings, it was discussed that acceptance was interpreted by some as negative and defeatist, while others insisted it was a vitally important outcome as it marks a starting point for change and improvement in psychological symptoms. What do you think about that?

At the consensus meetings, it was discussed that some people did not like the word “acceptance” as it had connotations of giving up and making do with tinnitus, while others felt that it was the correct and best word for the definition and the emotional coming to terms with tinnitus. What do you think about that? Is the word “acceptance” the right term for the outcome or would you prefer it to have a different name?

OPTIONAL: How do you feel "acceptance of tinnitus" is distinct from "sense of control"? In what ways are they different and how can we pick them apart?

At the consensus meetings, it was discussed that acceptance should include annoyance and irritability. What do you think about that? Do we think they both belong within the definition of acceptance?

It was also discussed that annoyance and irritability may fit within the other core outcomes of tinnitus intrusiveness, ability to ignore, or mood. Where do you think they fit best?

OR

At the consensus meetings, it was discussed that acceptance should include annoyance. What do you think about that? Do we think that belongs within the definition of acceptance?

It was also discussed that annoyance may fit within the other core outcomes of tinnitus intrusiveness, ability to ignore, or mood. Where do you think it fits best?

At the consensus meetings, it was discussed that acceptance should include irritability. What do you think about that? Do we think that belongs within the definition of acceptance?

It was also discussed that irritability may fit within the other core outcomes of tinnitus intrusiveness, ability to ignore, or mood. Where do you think it fits best?

*Definitions:*

*Annoyance: Noticing the sound of tinnitus is there and it feels like a nuisance*

*Irritability: Having a tendency to easily feel tense, on edge or agitated because of your tinnitus*

At the consensus meetings, it was discussed that acceptance should include coping. What do you think about that? Do we think that belongs within the definition of acceptance?

It was also discussed that coping may fit within the other core outcome of tinnitus intrusiveness. Where do you think it fits best?

*Definition:*

*Coping: Ability to deal with or handle tinnitus*

Thank you so much for all of your discussion, it has been really useful having your ideas and opinions to guide us in this difficult process of defining the core outcomes, and we appreciate the time you have dedicated to taking part. To summarise, it seems/we have decided/it seems we all agree [things decided to be added that the outcome should cover, changes to/issues with the original definition, any remaining debates or disagreements if definitely unresolvable]. Therefore, a new definition for the outcome acceptance of tinnitus may be […]

Poll: Do you agree with the summary and new definition?

1. Yes, it accurately summarises the discussion and I agree with the new definition
2. It accurately summarises the discussion but I do not agree with the new definition
3. No, it does not accurately summarise the discussion and I do not agree with the new definition

Thank you all for taking part. This discussion is now closed. If you were unable to take part while the discussion was open for any reason, or have any final comments that you want to add, you can send them to [hearing NIHR email address?]. We will not be able to change any of the discussion’s conclusions or changes made to the definition but we may use late comments to inform our decisions in the next stage of the research, and they may be included in the reporting of these discussions.

## Concentration discussion pack

The original definition of concentration is “ability to keep your attention focused”.

What do you like about this definition and what do you think could be changed or improved?

What does concentration mean to you?

Remembering that concentration has been chosen as a core outcome for sound-based interventions for tinnitus, what aspects of concentration would you expect a successful sound-based intervention to change?

What experiences and aspects of tinnitus would you want a questionnaire about concentration to ask about? Is there anything that, if it wasn’t mentioned, would make you feel like the questionnaire had failed and missed the point of concentration?

OR

What experiences and aspects of concentration would you want a questionnaire about tinnitus to ask about? Is there anything that, if it wasn’t mentioned, would make you feel like the questionnaire had failed and missed the point of concentration with tinnitus?

At the consensus meetings, it was discussed that concentration should include conversations and listening. What do you think about that? Do we think they both belong within the definition of concentration?

It was also discussed that conversations and listening may fit within the other core outcome of ability to ignore or tinnitus loudness. Where do you think they fit best?

OR

At the consensus meetings, it was discussed that concentration should include conversations. What do you think about that? Do we think that belongs within the definition of concentration?

It was also discussed that conversations may fit within the other core outcome of ability to ignore or tinnitus loudness. Where do you think it fits best?

At the consensus meetings, it was discussed that concentration should include listening. What do you think about that? Do we think that belongs within the definition of concentration?

It was also discussed that listening may fit within the other core outcome of ability to ignore or tinnitus loudness. Where do you think it fits best?

*Definitions:*

*Conversation: Effect of tinnitus (not hearing loss) on ability to listen, understand and take part in conversations*

*Listening: Effect of tinnitus on ability to understand somebody talking (e.g. TV and radio)*

OPTIONAL: How do you feel "ability to ignore" is distinct from "concentration"? In what ways are they different and how can we pick them apart?

At the consensus meetings, it was discussed that concentration should include impacts on work and impacts on social life. What do you think about that? Do we think they both belong within the definition of concentration?

It was also discussed that impacts on work and impacts on social life may fit within the other core outcomes of tinnitus intrusiveness or ability to ignore. Where do you think they fit best?

OR

At the consensus meetings, it was discussed that concentration should include impacts on work. What do you think about that? Do we think that belongs within the definition of concentration?

It was also discussed that impacts on work may fit within the other core outcomes of tinnitus intrusiveness or ability to ignore. Where do you think it fits best?

At the consensus meetings, it was discussed that concentration should include impacts on social life. What do you think about that? Do we think that belongs within the definition of concentration?

It was also discussed that impacts on social life may fit within the other core outcomes of tinnitus intrusiveness or ability to ignore. Where do you think it fits best?

*Definitions:*

*Impact on work: Effect of tinnitus on your ability to carry out work tasks or job roles*

*Impact on social life: Effect of tinnitus on the ability to take part fully in a group social gathering (e.g. at a restaurant, at the park or at a party)*

Thank you so much for all of your discussion, it has been really useful having your ideas and opinions to guide us in this difficult process of defining the core outcomes, and we appreciate the time you have dedicated to taking part. To summarise, it seems/we have decided/it seems we all agree [things decided to be added that the outcome should cover, changes to/issues with the original definition, any remaining debates or disagreements if definitely unresolvable]. Therefore, a new definition for the outcome concentration may be […]

Poll: Do you agree with the summary and new definition?

1. Yes, it accurately summarises the discussion and I agree with the new definition
2. It accurately summarises the discussion but I do not agree with the new definition
3. No, it does not accurately summarise the discussion and I do not agree with the new definition

Thank you all for taking part. This discussion is now closed. If you were unable to take part while the discussion was open for any reason, or have any final comments that you want to add, you can send them to [hearingnihr@nottingham.ac.uk](mailto:hearingnihr@nottingham.ac.uk). We will not be able to change any of the discussion’s conclusions or changes made to the definition but we may use late comments to inform our decisions in the next stage of the research, and they may be included in the reporting of these discussions.

## Ability to ignore discussion pack

The original definition of ability to ignore is “Ability to continue as normal as if tinnitus were not there”.

What do you like about this definition and what do you think could be changed or improved?

What does ability to ignore mean to you?

Remembering that ability to ignore has been chosen as a core outcome for sound-based interventions for tinnitus, what aspects of ability to ignore would you expect a successful sound- based intervention to change?

What experiences and aspects of tinnitus would you want a questionnaire about ability to ignore to ask about? Is there anything that, if it wasn’t mentioned, would make you feel like the questionnaire had failed and missed the point of ability to ignore?

OR

What experiences and aspects of ability to ignore would you want a questionnaire about tinnitus to ask about? Is there anything that, if it wasn’t mentioned, would make you feel like the questionnaire had failed and missed the point of ability to ignore with tinnitus?

At the consensus meetings, it was discussed that some people felt ability to ignore should reflect a change in the tinnitus itself which has made it easier to ignore, while other felt it should reflect a change in the individual person’s abilities which has made them better at ignoring their tinnitus. What do you think?

Which of these two version of ability to ignore do you think should be used, or is it possible to combine them? The aim is to have one definition and ultimately one measurement instrument for each core outcome domain. Does it help to consider that it has been chosen as a core outcome for sound-based interventions for tinnitus?

At the consensus meetings, it was discussed that some people did not like the part of the definition that states “continue as normal” as they felt using even a successful sound-based intervention would deviate from life as normal. What do you think about that? Is the definition of ability to ignore ok or does that part of the definition need to be removed or changed?

At the consensus meetings, it was discussed that some people felt that it was necessary for the definition to specify that it is their ability to ignore their tinnitus if/when it is unpleasant or intrusive, and not just all the time in all contexts. What do you think about that? Is the definition of ability to ignore ok or does that need to be added to the definition or changed in some way?

At the consensus meetings, it was discussed that ability to ignore should include annoyance. What do you think about that? Do we think that belongs within the definition of ability to ignore?

It was also discussed that annoyance may fit within the other core outcomes of acceptance of tinnitus, tinnitus intrusiveness, or mood. Where do you think it fits best?

*Definitions:*

*Annoyance: Noticing the sound of tinnitus is there and it feels like a nuisance*

At the consensus meetings, it was discussed that ability to ignore should include the impact of tinnitus on work, social life and individual activities. What do you think about that? Do we think they belong within the definition of ability to ignore?

It was also discussed that impact on work, social life and individual activities may fit within the other core outcomes of tinnitus intrusiveness and concentration. Where do you think they fit best?

*Definitions:*

*Impact on individual activities: Effect of tinnitus on your choice to engage in your individual interests or tasks (e.g. driving, reading, listening to music or watching TV). Not group activities*

*Impact on relationships: Effect of tinnitus on relationships with family and friends*

*Impact on social life: Effect of tinnitus on the ability to take part fully in a group social gathering (e.g. at a restaurant, at the park or at a party)*

*Impact on work: Effect of tinnitus on your ability to carry out work tasks or job roles*

At the consensus meetings, it was discussed that ability to ignore should include conversations and listening. What do you think about that? Do we think they both belong within the definition of ability to ignore?

It was also discussed that conversations and listening may fit within the other core outcome of concentration or tinnitus loudness. Where do you think they fit best?

OR

At the consensus meetings, it was discussed that ability to ignore should include conversations. What do you think about that? Do we think that belongs within the definition of ability to ignore?

It was also discussed that conversations may fit within the other core outcome of concentration or tinnitus loudness. Where do you think it fits best?

At the consensus meetings, it was discussed that ability to ignore should include listening. What do you think about that? Do we think that belongs within the definition of ability to ignore?

It was also discussed that listening may fit within the other core outcome of concentration or tinnitus loudness. Where do you think it fits best?

*Definitions:*

*Conversation: Effect of tinnitus (not hearing loss) on ability to listen, understand and take part in conversations*

*Listening: Effect of tinnitus on ability to understand somebody talking (e.g. TV and radio)*

In the other threads, it has been suggested that ability to ignore may overlap with quality of sleep, which has also been chosen as a core outcome domain. Does anyone have any thoughts on how these two outcome domains differ and ways they could be separated?

In the concentration thread, it came up often the difficulty to separate it conceptually from ability to ignore. How do you feel "ability to ignore" is distinct from "concentration"? In what ways are they different and how can we pick them apart? (they were both selected as core outcomes for sound-based treatments, and it is important for there to be no overlap between the outcomes in the core set as far as is possible.)

Thank you so much for all of your discussion, it has been really useful having your ideas and opinions to guide us in this difficult process of defining the core outcomes, and we appreciate the time you have dedicated to taking part. To summarise, it seems/we have decided/it seems we all agree [things decided to be added that the outcome should cover, changes to/issues with the original definition, any remaining debates or disagreements if definitely unresolvable]. Therefore, a new definition for the outcome acceptance of tinnitus may be […]

Poll: Do you agree with the summary and new definition?

1. Yes, it accurately summarises the discussion and I agree with the new definition

2. It accurately summarises the discussion but I do not agree with the new definition

3. No, it does not accurately summarise the discussion and I do not agree with the new definition

Thank you all for taking part. This discussion is now closed. If you were unable to take part while the discussion was open for any reason, or have any final comments that you want to add, you can send them to [hearing NIHR email address?]. We will not be able to change any of the discussion’s conclusions or changes made to the definition but we may use late comments to inform our decisions in the next stage of the research, and they may be included in the reporting of these discussions.
