# Supplementary material for: Defining Symptom Concepts in Chronic Subjective Tinnitus: Web-Based Discussion Forum Study
Source: Interact J Med Res. 2020 Jan 7;9(1):e14446. doi: 10.2196/14446 (PMC6996772; doi:10.2196/14446)
Supplement: Multimedia Appendix 5 [file ijmr_v9i1e14446_app5.docx]

# Progressing towards a common standard: using an online discussion forum to define concepts in chronic subjective tinnitus symptomatology

# Multimedia Appendix 5: Executive summaries and revised plain language definitions for each core outcome domain, including voting results for each discussion thread.

## Tinnitus intrusiveness

Executive summary: The original definition of *tinnitus intrusiveness* was "Noticing the sound of tinnitus is there and it is invading your life or your personal space". To summarise, you all described what intrusiveness meant to you and put it into your own words, suggested what a questionnaire should ask to accurately tap into it, and described what changes to intrusiveness you would want a treatment to deliver to be considered effective. These will all be very useful in future stages of the research.

We covered and discovered a number of key things:

Key issues with the definition:

- Noticing is not the right word – most people notice their tinnitus all the time and intrusiveness is something beyond that
- It was lacking the necessary negative connotations of being severely unwelcome and unwanted
- “Sound” should be pluralised as many people hear multiple sounds (I have changed this in all the suggested definitions for the poll as it was the opinion of the vast majority), while others felt you shouldn’t even specify the sounds so it can encompass all of tinnitus’s effects

Key conceptual issues:

- Whether intrusiveness is about the mere presence and awareness of the tinnitus, or about the negative effects of it on you and your life - the vast majority supported the latter and agreed that intrusiveness should be a measure of impact and effect
- Intrusiveness is incredibly individual – different people are affected by tinnitus in entirely different ways, and the definition, construct and measure of intrusiveness needs to allow for this although that is difficult to do in practice

Key decisions:

- Intrusiveness should include the impact domains (impact on individual activities/work/social life/relationships) although some do seem to belong within other core outcomes too (e.g. impact on work belonging in concentration) so they still need to be picked apart to prevent any overlap
- Intrusiveness should include tinnitus awareness and tinnitus unpleasantness as a foundation, but it goes far beyond that
- Intrusiveness may be related to wider quality of life, but that is beyond the remit of the outcome and doesn’t need to be in the definition, although it may inform how it can be measured
- Intrusiveness is closely related to several of the other core outcomes (concentration, ability to ignore, quality of sleep) especially sense of control, but it doesn’t seem to me like there is any significant risk of overlap from the points everyone has raised, just that they are complimentary concepts and there is a relationship between them
- Intrusiveness should NOT include coping as that probably belongs better within sense of control
- Most people did not have a strong opinion of whether intrusiveness should include annoyance and irritability, as these may fit either here or within mood.

Below is a full list of the revised definitions to be voted on in the poll. These are the most supported and most unique definitions suggested for *tinnitus intrusiveness* throughout the course of the discussion.

1. “Noticing that the sounds of tinnitus are invading your life or your personal space” (0%)

2. “Being acutely aware of the sounds of tinnitus and feeling that it is invading your life or your personal space” (5%)

3. “Being acutely aware of the sounds of tinnitus and it is changing your thoughts or actions” (5%)

4. “An unpleasant and overwhelming awareness of the sounds of tinnitus” (5%)

5. “The feeling that the tinnitus is overwhelming, dominating or invasive” (0%)

6. “The unacceptable and unwelcome interference of internal head and body noise heard only by the individual” (15%)

7. “The extent to which tinnitus invades your life, stresses you in daily situations and prevents you from doing things you want to do” (35%)

8. I cannot decide between at least two of the definitions, and will leave a post below to identify which ones I support (20%)

9. I do not like any of these definitions, and will leave a post below suggesting a new one that I prefer (5%)

## Sense of control

Executive summary: The original definition of *sense of control* was "Whether or not you feel you have a choice in how to manage the impact of tinnitus and feelings caused by tinnitus". To summarise, you all described what sense of control meant to you in your own words, suggested what a questionnaire should ask to tap into it, and described what changes to sense of control you would want a sound or psychology-based treatment to deliver to consider it effective. These will all be very useful in the next stages of the research.

We covered and discovered a number of key things:

Key issues with the definition:

- It needed something added to recognise that the choices and options available are effective/viable
- It needed to be more specific – we always have a choice how to respond, but it should refer to i. Free personal choice, ii. Learned strategies, iii. Available resources
- It needed to make it clearer that tinnitus is not a mental health issue, referring to all impacts, including emotional and practical

Key conceptual issues:

- Whether sense of control is about controlling the tinnitus itself or controlling the impact tinnitus has on you and your life
  - Majority felt the latter is most relevant and important, but that we should allow for controlling the tinnitus itself to be covered as well
- Is letting go of the need for control a part of sense of control itself, or would that fall under acceptance?
  - Although controversial and good points made on both sides, it seems agreement has now reached that it falls under acceptance? Please share your thoughts on this debate below if you have not already

Key decisions:

- the outcome domain “coping” should belong within sense of control
- “self-efficacy” is a part of sense of control
- Although the two outcomes are undeniably related, sense of control is distinct from “acceptance of tinnitus” because it is active and situational rather than passive or general, and about managing rather than letting go.

As a result of this discussion, with all of these conceptual issues and key decisions underlying the definition, the revised definition to be voted on is: "Feeling that you have effective options for managing the impacts of and feelings caused by tinnitus, through an understanding of your condition, learned strategies and/or available resources." (81.8%)

## Acceptance of tinnitus

Executive summary: The original definition of *acceptance of tinnitus* was "Recognising that tinnitus is a part of your life without having a negative reaction to it". To summarise:

Describing acceptance in your own words helped clarify the construct. Some of you shared personal experiences to demonstrate what acceptance can look like, while others discussed the definition of acceptance in psychological literature.

Suggestions were made as to what a questionnaire should cover, and what an effective psychology-based tinnitus treatment could change about acceptance. This put the construct in context and will help in later stages of the research identifying how concentration should be measured.

We have begun to sort out some areas of overlap with the other core outcomes (“annoyance” belongs under tinnitus intrusiveness, “coping” belongs under sense of control) and identify the differences between acceptance and sense of control (sense of control refers to the feeling achieved once a treatment or coping strategy is found that helps manage the tinnitus symptoms is found, and acceptance refers to the general feeling of being at peace with the tinnitus, no longer fighting against it, usually once a sense of control has been achieved). We will continue to discuss these in the upcoming discussions over the next 3-4 weeks.

Key things learned:

- Acceptance is an incredibly emotive outcome because of how difficult it is to achieve, and how it can seem entirely unrealistic and unachievable to those who are not there yet. We acknowledged the divided opinions of acceptance as giving up or the friction it can create between patients and practitioners. We discussed whether the definition was realistic and inclusive to all, and identified the need for questionnaires to be worded sensitively as they have the potential frustrate or upset.
- Some people did not like the word “acceptance” and advocated for “endurance”, “resilience” or “tolerance” instead. However the majority argued that these concepts are slightly different to acceptance with more negative connotations, and perhaps represented steps on the journey to total acceptance, and so that should remain the outcome that is being aimed for.

Two aspects of the definition were identified as unrealistic and needing change:

- It was agreed that there was nothing wrong with have a negative reaction to tinnitus sometimes - this is inevitable and suggesting it should never happen is unhelpful. We decided that the key thing is that you can recognise the negative reaction is only temporary, choose not to act on it, and bring yourself back to a place of feeling neutral towards the tinnitus.
- It was agreed that acceptance is not a linear process, and is an ongoing ever-changing battle, and will always be better on some days than others. We explored how this could be measured in a questionnaire with many good suggestions including asking about up to two week time period, keeping a diary, or asking about critical milestones.

As a result of the discussion, a new definition was suggested: recognising that tinnitus is part of your life, staying neutral towards it in both thoughts and actions, and understanding that occasional negative reactions are inevitable but will pass. This was liked generally but deemed too long. Below is the abbreviated version that received most support and so the revised definition to be voted on is: “Recognising that tinnitus is part of your life, and staying neutral towards it in both thoughts and actions” (84.9%)

## Concentration

Executive summary: The original definition of *concentration* was "ability to keep your attention focused". To summarise:

Concentration is understood by most to mean roughly the same thing. Describing it in your own words and sharing examples of tasks, activities and times that tinnitus affects your concentration helped to clarify the construct

Suggestions were made as to what a questionnaire should cover, and what an effective sound-based tinnitus treatment could change about concentration. This helped to put the construct in context and will help in later stages of the research identifying how concentration should be measured.

We have begun to sort out some areas of overlap with the other core outcomes (“conversations” and “listening” belong in ability to ignore, “impact on social life” belongs in intrusiveness) and identify the differences between concentration and ability to ignore (concentration is ability to focus on a specific task or activity that requires full attention, ability to ignore is ability to focus off of tinnitus all the time and in all situations). We will continue to discuss these in the upcoming discussions over the next 4-5 weeks

Key things we have learned:

- The amount that tinnitus affects concentration is task-dependent/environment-dependent. A variety of tasks and environments should be asked about separately in a questionnaire as they are expected to show a difference. We theorised as to what sort of tasks and environments make concentration more difficult (more technical or verbal, requiring higher-order skills, important, stressful, solitary or a simple sensory experience – driving is an exception due to some people’s tinnitus sound being similar to emergency vehicle sirens or engine sounds.)
- Concentration with tinnitus is not a yes or no question, but rather a spectrum. It is important to note the additional effort that is required to concentrate with tinnitus, and the fatigue or cognitive tiredness that results. This should be explored within a questionnaire as it may appear on the surface that an individual is managing to concentrate fine, overlooking this additional effort. This is also a key aim for improvement by a sound-based treatment.

There are three schools of thought on what changes to the definition are needed:

- something should be added to specify that the key issue is not concentrating on the tinnitus – e.g. ““the ability to keep attention focused off of tinnitus/on anything more than the tinnitus/without being distracted by tinnitus”
- something should be added but it shouldn’t specify tinnitus, as that gives it power and implies it is still the focus – e.g. “the ability to keep attention focused fully/ on the task at hand/ elsewhere/ with intention”
- Nothing should be added, as the current definition is fine and if it is too long or overcomplicated it will become meaningless

Therefore I'm not sure we will be able to please everyone, but the revised definition that received the most support during the discussion is: “The ability to keep your attention focused on whatever you wish” (100%).

## Ability to ignore

Executive summary: The original definition of *ability to ignore* was "Ability to continue as normal as if tinnitus were not there". To summarise, we have discussed the definition of ability to ignore, suggesting alternatives to improve it and describing what it means to you in your own words. Some of you suggested what a questionnaire should ask to tap into ability to ignore and described the changes you would want to see to consider a sound-based treatment effective.

We covered and discovered a number of key things:

Key issues with the definition:

- Many people didn’t like the word “normal” (what is normal anyway? Life with tinnitus will never be “normal” if using a sound-based treatment so an irrelevant and arguably unhealthy concept). The majority agreed that this word should be removed from the definition
- There was debate over whether the word “despite” should be used or “as if” (“despite” more realistic, “as if” more positive). Both seemed justified and a matter of personal preference, but the majority supported “as if”.
- Some people did not like the word “continue” (ability to ignore shouldn’t just be about when doing something, but also when just being e.g. to allow relaxation and peace). Suggestions were made to use the word “function” or “feel” but not enough people shared their views of this. Note: if you like the new definition below but would rather "function" or "feel" is used than “continue”, please vote agree to the definition and leave a post below explaining which word you would prefer

Key conceptual issues:

- There was some debate as to whether ability to ignore should reflect a change in the tinnitus or in the individual – the majority said either, neither or somewhere in between as it should reflect a change in perception of the tinnitus, with useful analogies of glasses to explain this.
- Some felt ability to ignore is not inclusive of those with severe tinnitus
- All of the core outcomes are intended to be measured on a spectrum as an aim for treatments to achieve, so everyone is included by definition
- No one made any suggestions as to how the definition or concept of ability to ignore could be improved to make it more inclusive
- Some people just did not support the outcome ability to ignore (because it goes against other domains and personal goals which support acceptance/adaption to tinnitus, and that it is disrespectful to tell people just to ignore their suffering)
- The core outcomes were chosen by consensus of a large number of people from a diverse group of patients, professionals and researchers. We cannot change the set of outcomes now and that was not the aim of the discussions, as outlined in the Guidelines and Ground Rules.

Key decisions:

- “Conversation” and “listening” should belong under ability to ignore.
- The “impact” domains can either all belong under tinnitus intrusiveness or be split between ability to ignore (impact on individual activities and impact on social life), concentration (impact on work) and mood (impact on relationships)
- Ability to ignore is distinct from concentration in that it is about focusing away from the tinnitus rather than focusing towards something, and is relevant to low demand tasks rather than high demand, complex tasks.
- Ability to ignore is distinct from quality of sleep in that it is a coping skill (how well can the individual cope with their tinnitus) rather than an area of impact (how bad is the tinnitus).

As a result of all the discussion, the new definition to be voted on that received the most support throughout is: “Ability to continue as if tinnitus were not there​” (84%)
